# Dicentric chromosomes are resolved through breakage and repair at their centromeres

**DOI:** 10.1007/s00412-023-00814-6

**Published:** 2024-01-02

**Authors:** Diana Cook, Stanislav G. Kozmin, Elaine Yeh, Thomas D. Petes, Kerry Bloom

**Affiliations:** 1 Department of Biology, University of North Carolina Chapel Hill, Chapel Hill, NC 27599-3280 USA; 2 Department of Molecular Genetics & Microbiology, Duke University School of Medicine, Durham, NC 27710 USA

**Keywords:** Centromere, Recombination, Dicentric chromosome, Chromosome breakage, DNA repair

## Abstract

Chromosomes with two centromeres provide a unique opportunity to study chromosome breakage and DNA repair using completely endogenous cellular machinery. Using a conditional transcriptional promoter to control the second centromere, we are able to activate the dicentric chromosome and follow the appearance of DNA repair products. We find that the rate of appearance of DNA repair products resulting from homology-based mechanisms exceeds the expected rate based on their limited centromere homology (340 bp) and distance from one another (up to 46.3 kb). In order to identify whether DNA breaks originate in the centromere, we introduced 12 single-nucleotide polymorphisms (SNPs) into one of the centromeres. Analysis of the distribution of SNPs in the recombinant centromeres reveals that recombination was initiated with about equal frequency within the conserved centromere DNA elements CDEII and CDEIII of the two centromeres. The conversion tracts range from about 50 bp to the full length of the homology between the two centromeres (340 bp). Breakage and repair events within and between the centromeres can account for the efficiency and distribution of DNA repair products. We propose that in addition to providing a site for kinetochore assembly, the centromere may be a point of stress relief in the face of genomic perturbations.

**Supplementary Information:**

The online version contains supplementary material available at 10.1007/s00412-023-00814-6.

## Introduction

Centromeres are enigmatic regions of the chromosome. Centromere DNA is essential for the fidelity of chromosome segregation but shares little DNA sequence homology throughout phylogeny. Kinetochore proteins that assemble on the centromere are highly conserved, indicative of the evolutionary pressure on a conserved mode of DNA-microtubule attachment. Paradoxically, centromere DNA and the centromere-specific histone H3 variant are among the more rapidly evolving sequences (Drinnenberg, et al. 2016; Kursel, et al. 2017; Padmanabhan, et al. 2008). In addition, centromeres and centromere repeats present difficulties to the processivity of DNA polymerase (Greenfeder, et al. 1992). As replication stress is a known driver of DNA double-strand breaks (DSBs), the persistence of replication pause sites in these regions suggests that DNA polymerase pausing may be important for aspects of kinetochore assembly. Pauses in replication through centromere DNA are intrinsic to organisms with either point or regional centromeres, reflecting difficulties in navigating through kinetochore DNA binding proteins and/or alpha-satellite sequences (Greenfeder, et al. 1992; Hodgson, et al. 2007; Kobayashi, et al. 2017; Lopes, et al. 2006; Romeo, et al. 2016). To avoid delays in cell cycle progression, the cell has devised a means to suppress the powerful DNA checkpoint in order to facilitate centromere DNA replication (Aze, et al. 2016; Kabeche, et al. 2018).

Centromere recombination is differentially regulated in mitosis versus meiosis. Crossovers between centromere-linked genes are suppressed in meiosis. However, gene conversion between centromere satellite repeats is not suppressed (Shi, et al. 2010; Talbert, et al. 2010). Gene conversion can propagate through the centromere in both mitosis and meiosis in budding yeasts (Liebman, et al. 1988; Symington, et al. 1988). Gene conversion of alpha satellites provides a mechanism to account for the homogenization of satellites observed in regional centromeres of mammalian cells (Henikoff, et al. 2015). Satellite sequences are found in both direct and inverted orientations and are potential sources of complex chromosome rearrangements (Altemose, et al. 2022; Nurk, et al. 2022). It has been proposed that a set of kinetochore proteins (CENP-S, X aka MHF1, MHF2) and the FACNM/Fml1/Mph1 helicase are responsible for crossover avoidance within the centromere, but allow for a high degree of gene conversion (Bhattacharjee, et al. 2013; Prakash, et al. 2009). Recombination-prone alpha-satellite sequences in the centromere are sources of stress that lead to chromosome breakage and rearrangement (Bloom, et al. 2017; Branzei, et al. 2010; McFarlane, et al. 2010). Centromere DNA metabolism, including replication fork pausing, regression and restarts, DSB break repair, and recombination, must be integrated into kinetochore assembly and function to understand the structural determinants of a centromere.

Regions of replication pausing, known as replication slow zones (RSZs), have been identified as chromosome-fragile sites (Cha, et al. 2002). These sites were identified in budding yeasts based on their sensitivity to the loss of Mec1, the ATR kinase. Upon traversal of an RSZ, the site of stalled replication fork progression is converted into a DSB in the absence of Mec1. Interestingly, yeast centromeres were not converted to sites of DSBs, suggesting that centromeres have the means to suppress the response to replication pausing (Aze, et al. 2016; Romeo, et al. 2016). The breakage of these fragile sites requires topoisomerase and condensin, independent of spindle tension, anaphase, and cytokinesis (Hashash, et al. 2012).

The behavior of chromosomes with two homologous centromeres on the same DNA strand (dicentric chromosome) provides a means to query the breakage and repair pathways required for resolution of the chromosome to a monocentric derivative. The transition from monocentric to dicentric function can be precisely controlled by the conditionally regulated centromere. The mechanism of dicentric chromosome breakage is often attributed to microtubule pulling forces. Pulling forces generated through kinetochore-mediated microtubule motion have been measured by several investigators. Direct measurement using calibrated optical traps with purified kinetochore reveals the stall force for kinetochore motion to be on the order of 5-7 pN (Akiyoshi, et al. 2010). Estimates from the in vivo dynamics of lacO-LacI-GFP fusions in the pericentromere reveal the chromatin springs to resist 4-6 pN (Chacon, et al. 2014). In vivo modeling reveals that thermal forces from the molecular bottlebrush exert 5-10 pN on the centromere masses (Lawrimore, et al. 2022). These forces are insufficient by several orders of magnitude to sever covalent bonds. It is much more likely that errors in DNA replication (e.g., fork regression), enzymatic sources such as nuclease cleavage or errors in topoisomerase II function, or cytokinesis are responsible for dicentric chromosome breakage (Hashash, et al. 2012; Lopez, et al. 2015; Zhang, et al. 2007). In addition, chromosomes that remain in the bud neck following cytokinesis are severed as the cell undergoes abscission (Guérin, et al. 2019; Lopez, et al. 2015). The force of cell wall growth is sufficient to break the DNA backbone.

If DNA breaks occur randomly between the two centromeres, then we expect a spectrum of deletion derivative chromosomes harboring variable amounts of DNA between the two centromeres. These deletion derivatives would break in subsequent cell cycles until such time as stable monocentric chromosomes arise in the population. Analysis of deletion derivatives reveals that in contrast to expectations, deletion derivatives come in two predominant genotypes, depending on the distance between the two centromeres (Cook, et al. 2021). The derivatives have either complete deletions of one of the two centromeres or contain both centromeres rearranged via reciprocal cross-over into linear and circular derivatives. Whole genome sequencing of 25 colonies revealed no additional deletions (Cook, et al. 2021) that would be expected where chromosomes undergo multiple breakage-fusion-bridge cycles.

Monocentric derivative chromosomes that have lost either CEN3 or GALCEN3 must experience a DSB within or near the centromere. Likewise, broken dicentric chromosomes repaired via reciprocal cross-overs between the 340 bp of centromere homology must expose a DSB within the region of homology to initiate crossing-over. In this study, we have examined the kinetics of DNA repair products following activation of a dicentric chromosome in wild-type and DNA repair mutants and observed reciprocal crossovers using single-nucleotide polymorphisms (SNPs) between the two centromeres to determine whether there are breaks within the centromere. Breaks at the centromere account for the kinetics and distribution of monocentric derivatives and raise new questions about how the cell maintains genome stability at the centromere.

## Results

### Kinetics of centromere recombination

**Fig. 1:**
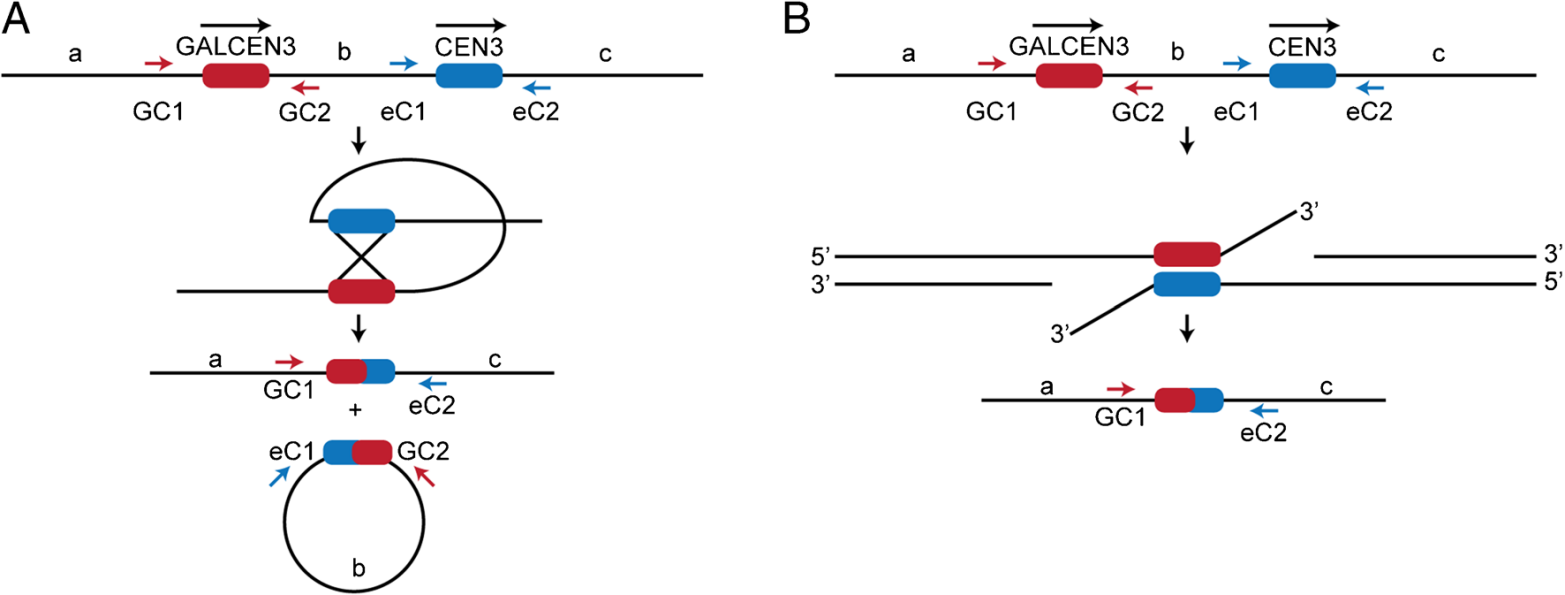
Centromere Homology Repair Pathways. **A** Schematic of the Reciprocal Crossover Event (RCO) that is the dominant repair pathway in dicentric chromosomes with 46.3 kb between the two centromeres. The two centromeres (GALCEN3 and CEN3) are in direct orientation. Primers used to amplify parental and recombinant products are marked. Recombinant centromeres arise in the repair products; GC1-eC2 and eC1-GC2 are a result of the two RCO events. **B** Schematic of the Single-Strand Annealing Event (SSA). This event yields a recombinant centromere that can be identified with GC1-eC2 primers

Activation of a conditionally functional dicentric chromosome in which the two centromeres are homologous results in the generation of monocentric derivatives. Homology-based repair pathways include reciprocal crossing over (RCO, Fig. 1A) and single-strand annealing (SSA, Fig. 1B) when the centromeres are oriented as direct repeats (Brock, et al. 1994). Repair efficiency can be estimated from 60 to 70% of cells that survive dicentric chromosome activation (Cook, et al. 2021). High cellular viability together with repair through centromere homology leads to the question of whether the centromere is a preferred site of recombination.

**Fig. 2:**
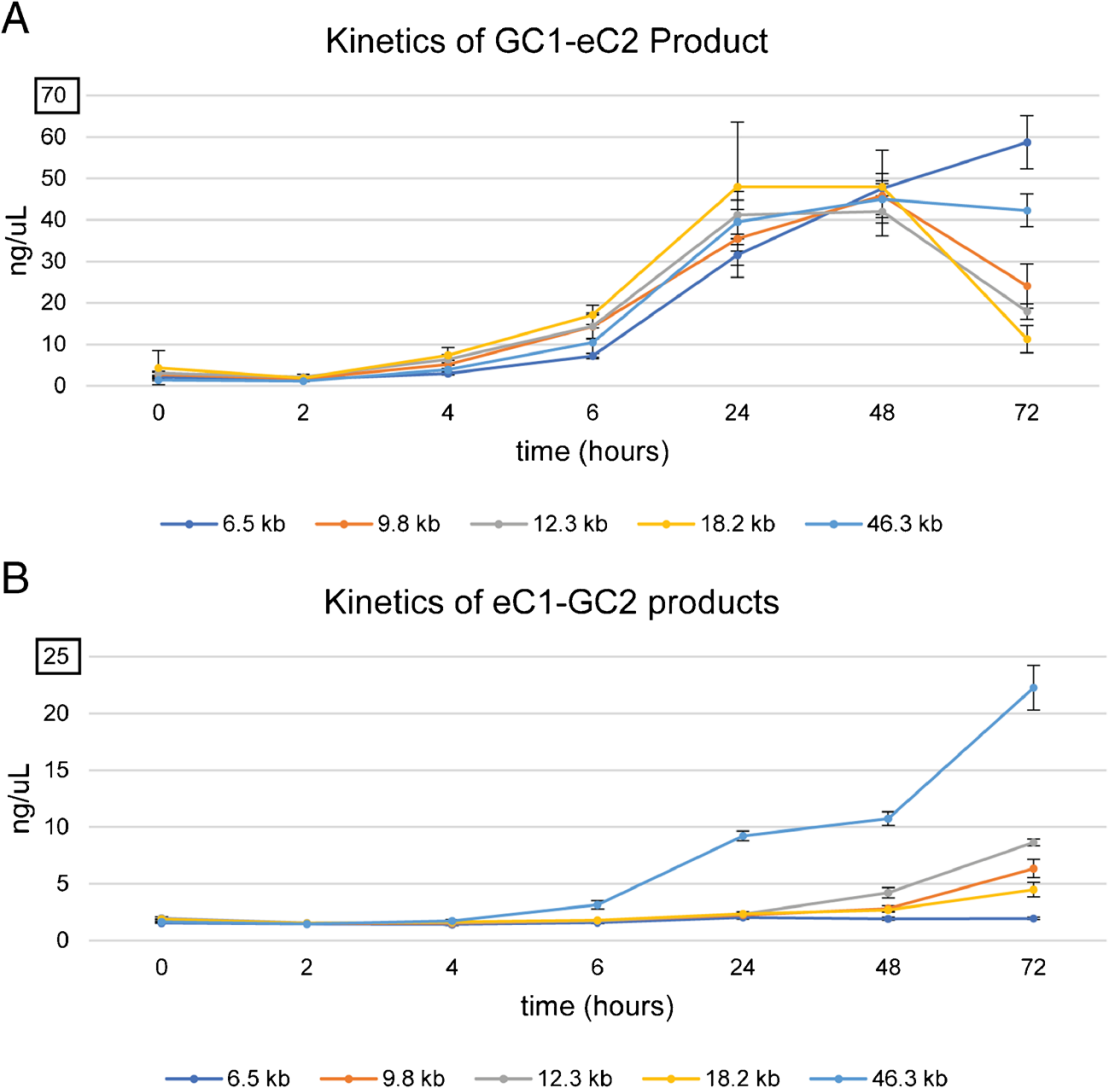
Recombinant products after dicentric chromosome activation. **A** Fluorimetry quantitation of recombinant PCR product GC1-eC2 in strains with GALCEN3 inserted 6.5, 9.8, 12.3, 18.2, and 46.3 kb away from the endogenous CEN3, after switching carbon source from galactose to glucose (GALCEN3 activation) and growing for indicated times. From 0 to 6 h, the kinetics of GC1-eC2 product generation are similar for all strains (see Fig. S2). The GC1-eC2 product is the result of RCO between the two centromeres (Fig. 1A) and the non-reciprocal SSA event (Fig. 1B). The time courses represent data from three independent cultures. *N*=9 for each data point, error bars are the standard error of the mean. **B** Fluorimetry quantitation of PCR product eC1-GC2. Note the difference in *y*-axis scaling compared to A, eC1-GC2 is not stoichiometric with GC1-eC2. The 46.3 kb dicentric is the only strain with markedly elevated eC1-GC2 product after 72 h due to selection for the RCO event at this CEN-CEN distance. The time courses represent data from three independent cultures. *N* = 9 for each data point, error bars are the standard error of the mean

To examine the kinetics of repair, we quantitated the appearance of recombinant products following activation of the Gal1-regulated centromere (GALCEN3) at varying distances from the endogenous CEN3 (Fig. 2). The parental centromeres, CEN3 and GALCEN3, were detected through PCR with eC1-eC2 (endogenous centromere) and GC1-GC2 (Gal1-regulated centromere) (Fig. S1) and the recombinant products with eC1-GC2 (endogenous Cen to GalCen) and GC1-eC2 primers (GalCen to endogenous Cen) (Fig. 2). Fluorimetry quantitation of recombinant products shown in Fig. 2 reveals these products to be far from stoichiometric (note scale bars on *y*-axis in Fig. 2). Both recombinant products increase over time from 2 to 24 h (Fig. 2). Time points from 2 to 6 h are reflective of initial events following dicentric chromosome activation, while accumulation at later time points (24–72 h) reflect selective advantages in growth or cell cycle progression. There is a 2-fold variation in the early time points for the GC1-eC2 and eC1-GC2 products (at 6 h), but there is no relation to inter-centromere distance (18.2 kb > 12.3 and 9.8 > 46.3 > 6.5) (Fig. S2).

The eC1-GC2 product is significantly reduced relative to GC1-eC2 (Fig. 2). In addition, the eC1-GC2 product is most evident in the 46.3 kb dicentric (from 6 to 72 h, Fig. 2B), versus in the pericentric dicentrics (18.2, 12.3, 9.8, and 6.5). The marked distance-dependent relationship in the eC1-GC2 product is consistent with the preferential generation of the ring and rod monocentric derivatives found in the 46.3 kb dicentric. Reciprocal crossover (RCO) between GALCEN3 and CEN3 is the preferred repair event in dicentric chromosomes with 46.3 kb between the two centromeres due to the selection for an essential gene (*NFS1*, 20 kb from *CEN3*) between the two centromeres (Cook, et al. 2021).

### Distinguishing homologous recombination pathways in dicentric chromosome repair

**Fig. 3:**
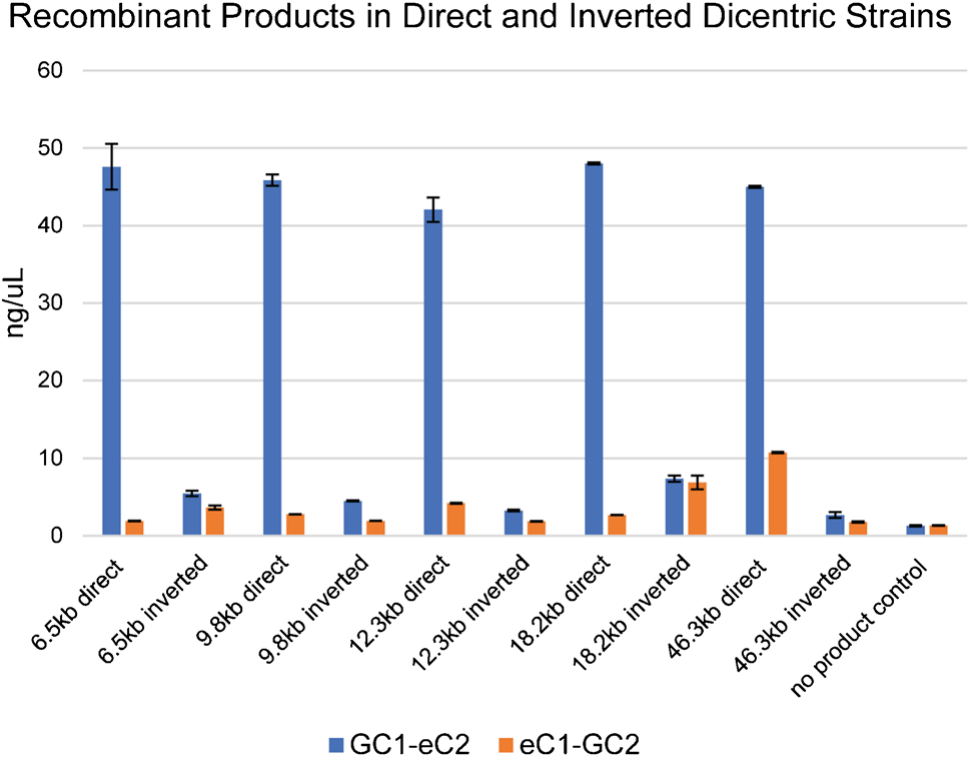
Stoichiometry of recombinant products as a function of position and orientation of the conditional centromere**.** Fluorimetry quantitation of GC1-eC2 and eC1-GC2 recombinant products after 48 h on glucose in strains with GALCEN3 inserted 6.5, 9.8, 12.3, 18.2, and 46.3 kb away from the endogenous CEN3, in both the direct orientation (Fig. 1A) and the inverted orientation, where GALCEN3 is flipped relative to the endogenous centromere. In dicentrics with centromeres in the direct orientation, the GC1-eC2 product (the results of RCO and non-reciprocal SSA) dominates the eC1-GC2 product, which is only produced through RCO. In strains with inverted oriented centromeres, SSA events lead to iso-dicentrics that rearrange in subsequent divisions (see text) and therefore do not accumulate in the population. The time courses represent data from three independent cultures. *N* = 9 for each data point except no product control where *n* = 3, error bars are standard error of the mean

The disparity in stoichiometry between the recombinant products is indicative of the multiple pathways to generate monocentric derivative chromosomes. To investigate the preferred DNA repair, we used quantitative analysis of the PCR products in strains with the two centromeres oriented as direct repeats (shown in Fig. 1) or inverted repeats (inverse of GALCEN3 in Fig. 1). Quantitative analysis was performed at the 48-h time point to ensure ample time to observe the recombinant products, but prior to bias due to growth selection. The GC1-eC2 recombinant product is the dominant event (~10-fold) in all dicentric locations where the two centromeres are in the direct orientation (dark blue column, direct orientation, Fig. 3). The presence of the GC1-eC2 product is predicted from a double-strand break between the two centromeres, followed by resection and single-strand reannealing of the 340 bp shared between the two centromeres (see Fig. 1B). The GC1-eC2 product would also arise following break-induced repair via the invasion of one centromere into the second, followed by DNA synthesis (break-induced repair, BIR). In a BIR event, a break at GALCEN3 followed by resection is followed by a single-strand invasion of the GALCEN3 into the endogenous centromere, where the invaded strand serves as the template for DNA synthesis. Either of these non-reciprocal pathways leads to stable monocentric derivatives lacking intervening DNA, the length of which depends on the distance between the two centromeres.

### Recombination between centromeres oriented as inverted repeats does not generate stable monocentric derivatives

The recombinant products in strains with inverted-orientation centromeres (GALCEN3 inverted relative to CEN3) are drastically different in yield relative to the direct orientation (Fig. 3). The major difference is the reduction of the GALCEN3-CEN3 (GC1-eC2) product (dark blue column, Fig. 3). Reciprocal cross-over between inverted centromere repeats generate a dicentric derivative chromosome with an inversion of DNA between the two centromeres, or broken fragments with one telomere and one broken end (the right and left chromosomal arms, respectively). SSA between centromeres in the inverted orientation results in iso-dicentric chromosomes through intra-chromosomal rearrangement or truncated chromosomes due to fold-back structures (Ramakrishnan, et al. 2018). In either case, stable monocentric derivatives are not generated. Rearrangement chromosomes containing the GC1-eC2 product from strains with inverted centromeres will continually rearrange until events arise that generate the monocentric derivative. The differences in the yield of recombinant products in the direct vs inverted dicentric strains can be accounted for by the inability of SSA or BIR events to yield stable monocentric derivatives when the regions of centromere homology are inverted with respect to one another.

**Fig. 4:**
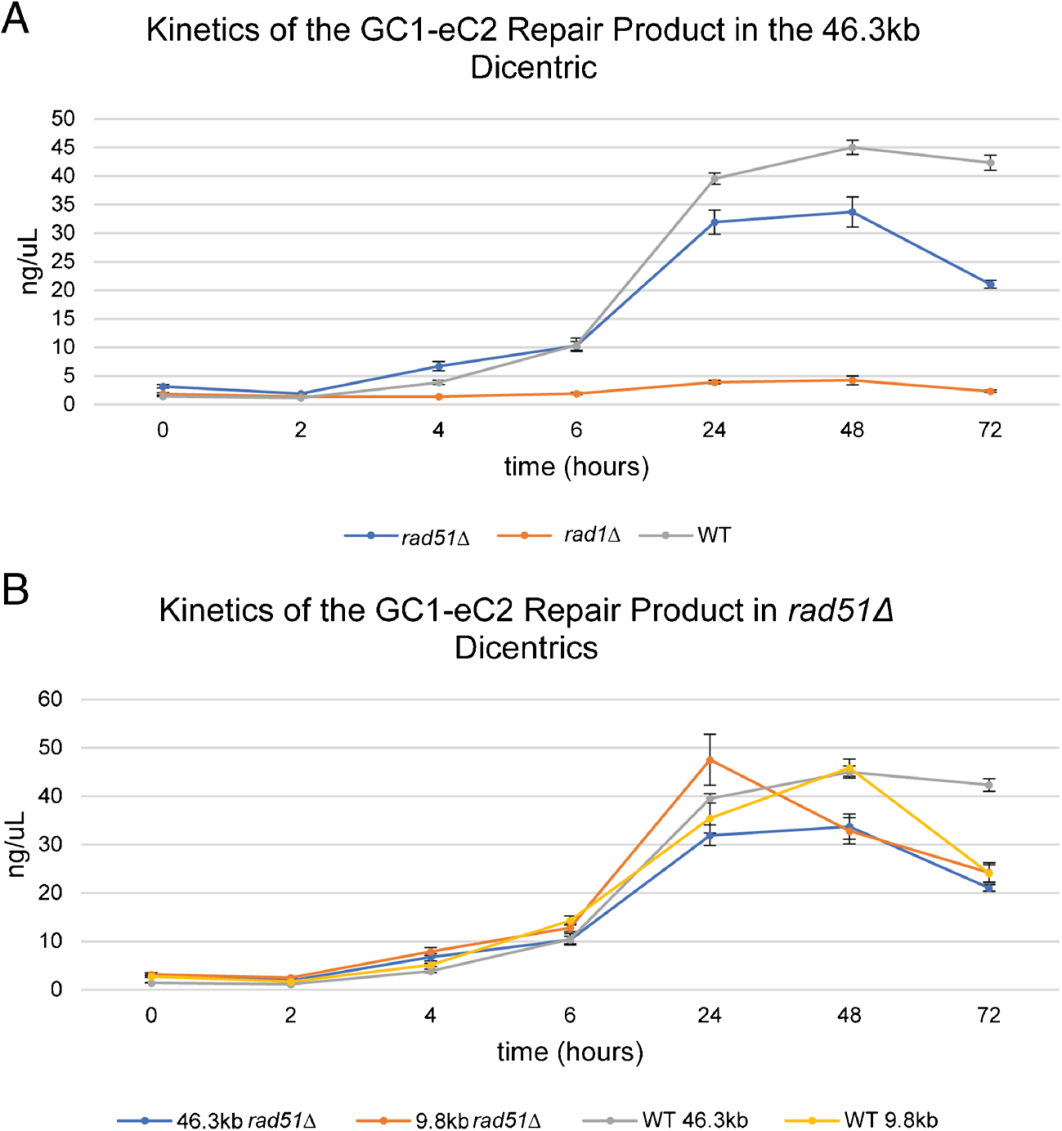
The GC1-eC2 Recombinant product is dependent upon Rad1 but not Rad51. **A** Fluorimetry quantitation of the recombinant PCR product GC1-eC2 in the 46.3 kb dicentric, WT, *rad51Δ*, and *rad1Δ*. WT data is from Fig. 2. The GC1-eC2 recombinant product is reduced in *rad1Δ. N* = 9 for each data point. Error bars are standard errors of the mean. Cell viability is reduced in the 46.3 kb *rad51Δ* strain, (45.1 ± 2.0%) compared to WT (61.0 ± 2.1%) (Cook et al., 2021), student’s *t*-test *p*-value < 0.01. Cell viability is not reduced in the 46.3 kb *rad1Δ* (54.5 ± 1.9%) compared to WT (61.0 ± 2.1%) (Cook et al., 2021), student’s *t*-test *p*-value > 0.01. Error is the standard error of the mean, *n* = 9 for each viability assay. **B** Fluorimetry quantitation of the recombinant PCR product GC1-eC2 in the 46.3 kb and 9.8 kb dicentrics, WT, and *rad51Δ.* WT data is from Fig. 2. GC1-eC2 is unaffected in *rad51Δ*. *N* =9 for each data point. Error bars are standard errors of the mean. The 9.8kb *rad51Δ* dicentric strain exhibits reduced viability (48.3 ± 1.1%) compared to WT (62.4 ± 1.2%) (Cook et al., 2021), student’s *t*-test *p*-value < 0.01. Error is the standard error of the mean, *n* = 9 for each viability assay

SSA requires the action of single-strand endonuclease RAD1/RAD10 to process the non-homologous tails flanking the region of homology (Fig. 1B) but does not require the product of RAD51, which is required for strand invasion. To investigate the contribution of SSA to dicentric repair, we examined the kinetics of recombinant products in the dicentric chromosomes with centromeres in the direct orientation and separated by 46.3 kb in *rad1Δ* and *rad51Δ* mutants (Fig. 4A). The yield of GC1-eC2 product is dependent on Rad1 and independent of Rad51 (Fig. 4A), consistent with a non-reciprocal single-strand annealing (SSA) event dominating the repair kinetics when centromeres are in the direct orientation (Fig. 3) (Brock, et al. 1994); (Thrower, et al. 2003).

The predominance of non-reciprocal homologous recombination in strains with the direct orientation provides the opportunity to estimate the site of breakage between the two centromeres. The requisite for SSA is the processing of DNA through 5′-3′ resection until single strands from each centromere become available for annealing with one another. The kinetics of SSA along dicentric chromosomes with very different centromere distances will distinguish whether breaks occur at random between the two centromeres, or whether there might be preferred break sites. These experiments were performed in the absence of Rad51 to rule out breaks within the centromere that could generate a distance-independent repair product via BIR. We examined the kinetics of the GC1-eC2 recombinant product in *rad51Δ* mutants in dicentric chromosomes with centromeres separated by 9.8 kb or 46.3 kb, respectively (Fig. 4B). The kinetics are comparable in the two strains from 0 to 6 h (Fig. 4B) and within the range of variation observed in wild-type strains in which there is no progression in the amount of product vs inter-centromere distance (Fig. 2). Thus, SSA is the predominant homology-based repair pathway responsible for the conversion of the dicentric to a monocentric deletion derivative (Figs. 3 and 4). The similarity in the kinetics of the GC1-eC2 products between centromeres whose distances range from 6.5 to 46.3 kb points toward the possibility that the initiating break sites are not randomly distributed between the two centromeres, rather they are biased toward the centromeres as reported by Song et al. (2013).

### Kinetics of single-strand annealing is distance-independent

Quantitative analysis of PCR products reveals that the kinetics of accumulation of the GC1-eC2 SSA product from 2 to 6 h on glucose is largely independent of the distance between the centromeres (Figs. 2 and S2). Considering the 7-fold change in distance (6.5 to 46.3 kb) and the rate of resection (3–4 kb/hr) (Yan, et al. 2019), we expected to see a difference in kinetics from 1 to 5 h (1 h for the 6.5 kb to 5 h for the 46.3 kb dicentric chromosome, respectively). Instead, the kinetics are comparable from 0 to 6 h (Figs. 2 and S2).

**Fig. 5:**
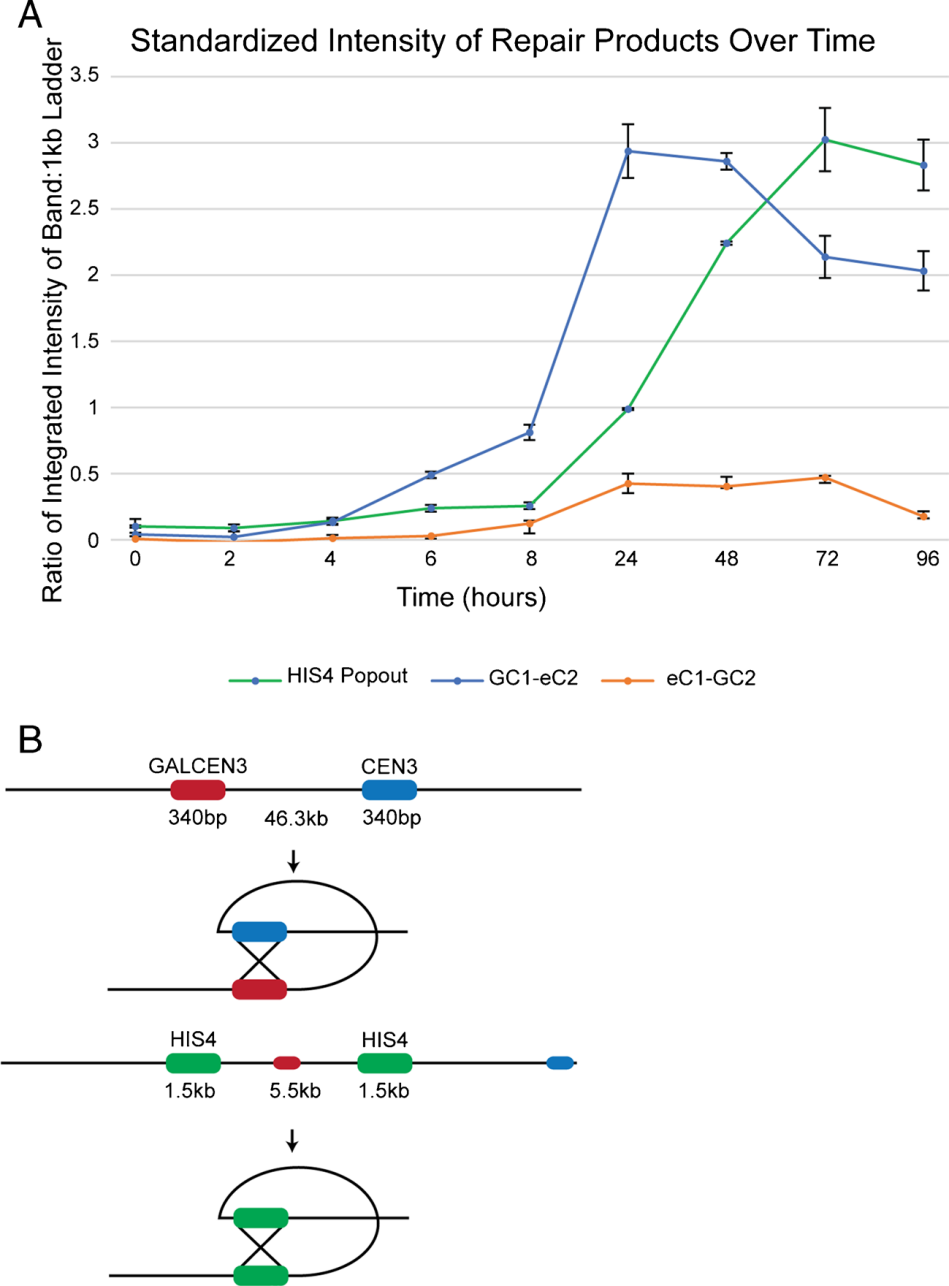
Kinetics of HIS4 vs CEN3 recombination. **A** Measurements of band intensity of recombinant PCR products in two different 46.3 kb dicentric strains. Strains are diagrammed in (**B**). Recombination between the two HIS4 repeats is kinetically delayed compared to recombination between CEN3 and GALCEN as measured by the GC1-eC2 product (blue line). The RCO product, eC1-GC2 (orange line), appears at the same time at the HIS4 rearrangement (green line) 24 h after activation, whereas GC1-eC2 appears at 4–6 h. There is a temporal bias toward CEN-CEN recombination that is not explained by homology or distance, since the HIS4 strain has more homology and shorter distance between repeats, which should facilitate homologous recombination at a faster rate than that between two distant centromeres. This bias could be the result of breaks within the centromere as opposed to random breaks between the two repeats. *N* = 2 for each data point. **B** Schematics of the two different dicentric chromosome constructs used in this assay. One strain has GALCEN3 (red) positioned 46.3 kb away from the endogenous CEN3 (blue) in the direct orientation. These identical centromeres only have 340 bp of homology to facilitate homologous recombination. The other strain has an identical dicentric construction but has a duplication of 1.5 kb of the HIS4 gene, with the repeat on the other side of the GALCEN3 sequence. The two HIS4 repeats are separated by 5.5 kb. Upon dicentric activation, recombination occurs via the HIS4 repeats

These data point to an initiating double-strand break or single-strand nick for DNA repair in the region of centromere homology rather than random breakage between the two centromeres. Prior studies have also pointed to the rapid appearance of the SSA repair product following the activation of the dicentric chromosome. Brock and Bloom (1994) observed the SSA product at 2.5–5 h following the growth of glucose. In contrast, the appearance of an SSA event via direct *HIS4* repeats was not apparent until 12–24 h (Brock, et al. 1994). In these dicentric chromosomes, a 1.56-kb region of the 5′ end of the *HIS4* gene is duplicated, with the repeats on either side of the GALCEN3 sequence. Activation of the dicentric stimulates non-reciprocal exchange between the *HIS4* repeats, and the resulting product was observed at 12–24 h (Brock, et al. 1994). To confirm and extend these results, strains containing the dicentric chromosome with centromeres in the direct orientation and flanked by *HIS4* repeats (Fig. 5B) were transferred to glucose, and the recombinant products were identified by PCR over time. As previously found, the centromere recombinant product (GC1-eC2) was observed by 4–6 h growth on glucose. In contrast, the *HIS4* rearrangement was observed at 24 h (Fig. 5A). The centromeres are 46.3 kb from one another and share 340 bp of homology, while *HIS4* repeats are 5.5 kb from one another and share 1.56 kb of homology. These data indicate that random breaks between repeats are unlikely to be initiating events responsible for centromere exchange. In contrast, if breaks are generated within the centromere, the prediction would be that a few hundred bp of resection will expose the two single-stranded centromeres for annealing, leading to rates of SSA independent of centromere–centromere distance. Alternatively, a DSB in one centromere may initiate a non-reciprocal recombination event with the unbroken centromere such as those observed in break-induced replication (Liu, et al. 2022).

### Single-nucleotide polymorphisms in the centromeres reveal reciprocal crossover (RCO) events following dicentric chromosome activation

**Fig. 6:**
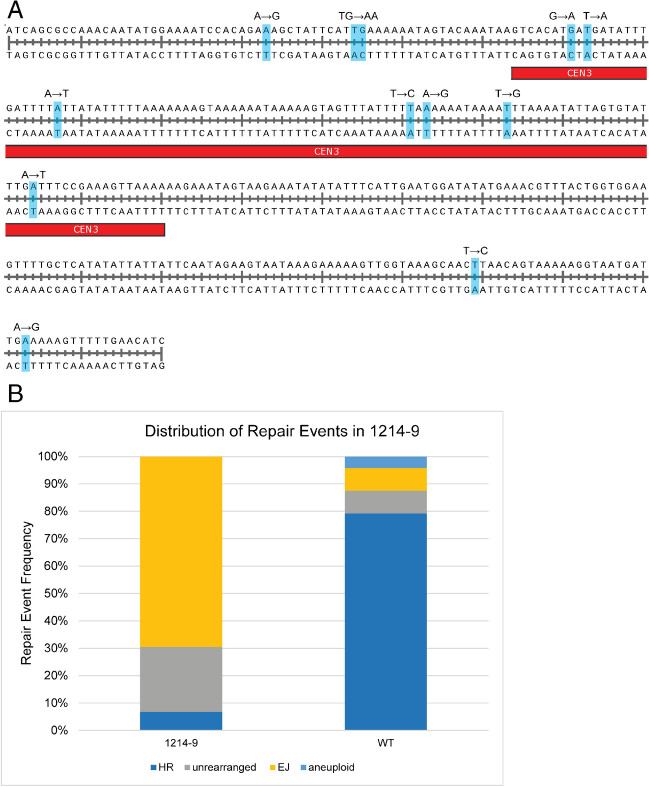
Centromere SNPs cause a shift in recombinant products. **A** Diagram of the CEN3 sequence derived from s288C with single-nucleotide polymorphisms marked in light blue, at positions 33, 44-45, 74, 76, 87, 131, 133, 144, 164, 299, and 323. The divergent CEN3 sequence is derived from YJM789 and contains 12 SNPs, 7 of which are within the 117-bp core centromere. The CEN3 and flanking bases shown here are the 340-bp that are homologous between CEN3 and GALCEN3. These SNPs can be used to analyze recombinant products on a granular level. **B** Recombinant endpoint analysis of a strain containing the s288C-derived GALCEN3 at 46.3 kb and the YJM789-derived CEN3 at the endogenous position (1214-9). Single colonies were analyzed by PCR for CEN3 (eC1-eC2), GALCEN3 (GC1-GC2), and the two recombinant products (GC1-eC2 and eC1-GC2). HR was defined as both recombinant products present and no parental products. SSA was defined as GC1-eC2 only. Unrearranged was both parental products and no recombinant products. End-joining was characterized by a deletion in one of the two centromeres and no other recombinant products. Aneuploid events contained a recombinant product and one or both unrearranged parental products. The pattern of repair is dramatically shifted from 79% homologous recombination in the WT to 69% end-joining. A small fraction (7%) are HR events, with the remainder (24%) in the “unrearranged” category. *N* = 24 for WT (no SNPs) (Cook et al., 2021) and *n* = 59 for 1214-9 (12 SNPs). The glu/gal viability for 1214-9 is 55.9 ± 1.3% (student’s *t*-test compared to WT, *p*-value > 0.01). Error is the standard error of the mean, *n* = 9 for viability assay

The initiating event for homologous recombination requires the invasion of a single-strand of DNA into a site of homology. The extent of homology between the two centromeres in the dicentric chromosome is 340 bp (Fig. 6A). Assuming a random distribution of breaks between the two centromeres, we would expect breaks at the centromere proper to represent less than 1% of the total inter-centromeric length (340/46,300 = 0.7%) in the 46.3-kb dicentric strain. The high viability (60–70%) of cells following dicentric chromosome activation (Brock, et al. 1994; Cook, et al. 2021) and the high fraction of survivors containing two reciprocal recombinant products (79% of survivors of the 46.3-kb dicentric contain GC1-eC2 and eC1-GC2 products) (Fig. 6B) reveals a discrepancy between the purported random distribution of breaks and the distribution of recombinant products in the survivors.

To directly test the hypothesis that recombination initiated within the centromere triggers the RCO event, we introduced single-nucleotide polymorphisms (SNPs) into one of the two centromeres (Fig. 6A). A centromere from yeast strain YJM789 (Wei, et al. 2007) was introduced in place of CEN3 at its endogenous location in chromosome III (114,300 bp) in a strain derived from s288c. The second centromere (GALCEN3 68,000 at *HIS4*) was derived from s288c. The sequences of the two centromeres are shown in Fig. 6A. There are 12 SNPs within the 340-bp repeats, (33 bp, A/G; 44–45 bp, TG/AA; 74 bp, G/A; 76 bp, T/A; 87 bp, A/T; 131 bp, T/C; 133 bp, A/G; 144 bp, T/G; 164 bp, A/T; 299 bp, T/C; 323 bp, A/G). The 117-bp CEN3 spans 64–180 (inclusive) and has seven of the 12 SNPs.

Strains containing SNPs between the two CEN3s on chromosome III exhibited viability indistinguishable from strains with identical CEN3s (55.9 ± 1.3% viability glucose/galactose (student’s *t*-test compared to WT, *p*-value > 0.01). In contrast, the distribution of products in the surviving colonies was shifted between the two strains. With a dicentric strain in which the GALCEN3 is identical to the native CEN3 and the two centromeric sequences are located 46.3 kb apart (DCY1214.1), most (79%) of the survivors in cells grown in glucose contain reciprocal recombinants, one linear chromosome with a deletion and a circular derivative with the deleted sequences (WT, Figs. 1A and 6B (Cook, et al. 2021). In strains with 12-centromere SNPs (strains DCY1214-9 and DCY1214-21), the surviving population is comprised of 69% end-joining events in which one of the two centromeres has been deleted and 24% events in which the two centromeres remain in the parental configuration (unrearranged, Fig. 6B). The reduction in the rate of crossovers in the strain in which the centromeres have 12 SNPs (resulting in 97% identity) is not unexpected. Datta et al. (1997) showed that repeats that were 99% identical had a rate of homologous recombination that was about 10-fold less than repeats that were 100% identical. This reduction is caused by the action of the DNA mismatch repair system reversing heteroduplexes that have mismatches. About 7% of the events in the survivors of the activation of the dicentric chromosomes with heteroallelic centromeres contained the reciprocal crossover products (Fig. 6B).

The class of unrearranged centromeres likely reflects a different mechanism. There are several Ty elements on chromosome III (at 1kb, 84 kb, 149 kb, and 169 kb). An alternative pathway toward the generation of monocentric derivative chromosomes is recombination events between Ty1 elements that flank the centromere at positions 84 kb and 149 kb. In cells with centromeres in the inverted orientation (Hill, et al. 1989), or cells with non-homologous centromeres (Surosky, et al. 1985) in the same chromosome, the major products were a linear 65-kb monocentric deletion derivative and the reciprocal 65-kb ring chromosome, each with one of the unrearranged parental centromeres (Hill, et al. 1989; Surosky, et al. 1985). It is possible that the events labeled “unrearranged” in the strain with the heterozygous SNPs contain these Ty rearrangements. The introduction of SNPs between the two centromeres thus reduces the rate of CEN–CEN recombination and increases the frequency of other types of events.

To assess the molecular nature of recombination events between the two centromeres, we sequenced centromeres from cells harboring the RCO event. Single colonies were isolated as detailed in the “Materials and methods” section, and RCO cells were identified as individuals with only centromere recombinant products (eC1-GC2 and GC1-eC2 products, Figs. 1A and 6B, HR) and no parental products or end-joining events. Products from 19 single-colony isolates were sequenced to determine the recipient and donor strands and the extent of gene conversion.

A reciprocal recombination event can result from a crossover anywhere within the 340-bp duplicated region containing the centromeres. Although from Fig. 6B, one might expect that sequencing of the linear and circular products would reveal two recombinant junctions, we can only detect such junctions if the recombination event occurs between heterozygous SNPs. Thus, any event that occurs between the start of the homology and the SNP at position 33 bp or between the SNPs at position 323 bp and the end of the homology will not result in a detectable recombinant junction. In addition, many previous studies in yeasts and other organisms (Symington, et al. 2014) have shown that reciprocal crossovers are usually associated with a non-reciprocal exchange of information (gene conversion) located adjacent to the DSB that initiates the event.

**Fig. 7:**
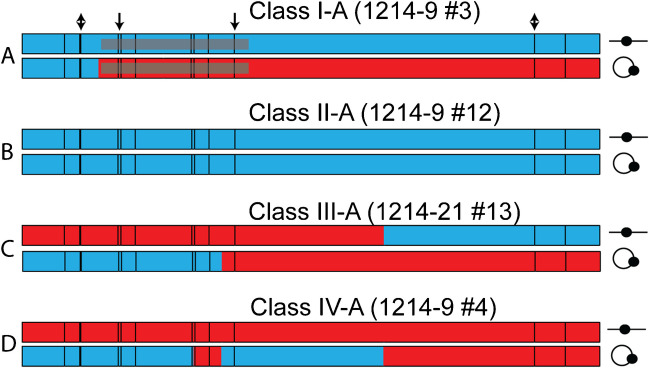
Four classes of reciprocal recombinants resulting from the resolution of a dicentric. Each pair of lines represents independent reciprocal recombinant products with the linear product (line plus dot) on the top and the circular product (circle plus dot) on the bottom. The length of each map is 340 bp which is the amount of DNA duplicated in generation of the dicentric. The contributions of the s288c and YJM789 sequences are shown in red and blue, respectively. Vertical black lines inside the rectangles indicate the positions of heterozygous SNPs. The centromeric sequences are 117 bp of the 340 bp repeat (Fig. 6). The SNPs within the centromeres at the boundaries are shown with black arrows and the SNPs at the boundaries of the flanking sequences are shown as double-headed arrows. Maps of all of the sequenced recombinants are in Fig. S3

Examples of the patterns of SNPs from the two different reciprocal recombinants are shown in Fig. 7. We divided the 19 analyzed recombinants into the following four classes: Class I (Fig. 7A 8 of 20), one chromosome with the parental arrangement of SNPs and one chromosome with a recombinant product; Class II (Fig. 7B 2 of 20), two chromosomes with centromeres with the parental (either both from one parent or one from each parent) configuration of SNPs; Class III (Fig. 7C, 5 of 20), both chromosomes with recombinant configurations of SNPs; and Class IV (Fig. 7D, 4 of 20), complex events in which one chromosome has two or more recombination breakpoints. In addition to the events depicted in Fig. 7, all of the sequenced events are shown in Fig. S3. For the recombinant products that have SNPs derived from only one of the centromeres, we infer that the breakpoint on this chromosome occurred in the regions of the duplication that did not contain heterozygous SNPs (proximal to the SNP at position 33 bp or distal to the SNP at position 323 bp) since all of the recombinant chromosomes had the PCR products expected for reciprocal exchanges (Fig. 6B). Seventeen of the 19 isolates had at least one detectable recombination breakpoint between polymorphisms. Of the 30 transitions between YJM789- and s288c-associated SNPs, 14 were within the centromeres, 11 were ambiguous (transition between a centromeric SNP and a flanking SNP), and 5 were unambiguously in the regions flanking the centromeres.

**Fig. 8:**
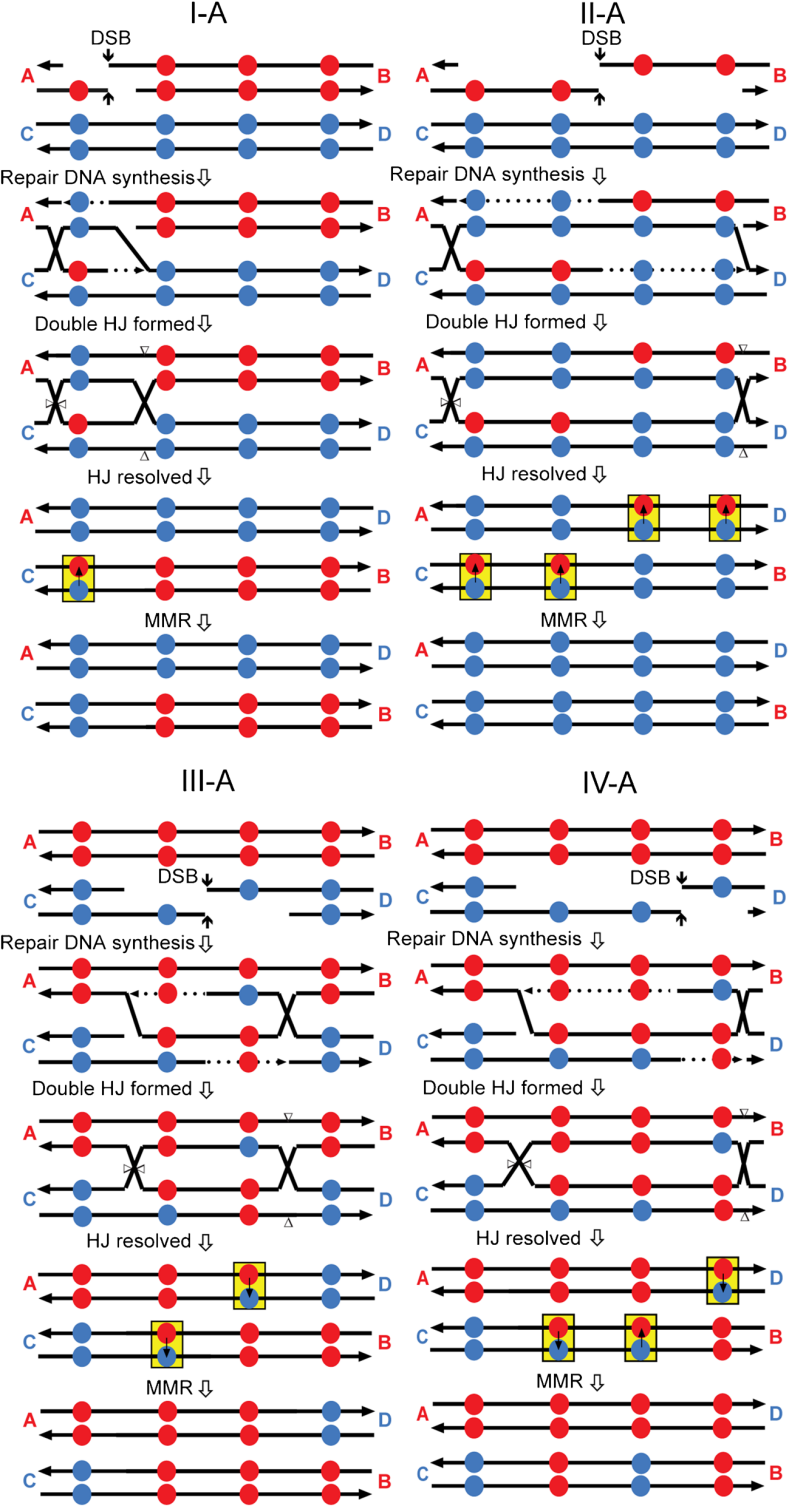
Mechanisms for generating the recombinant classes (classes I-A, II-A, III-A, and IV-A). DNA strands are represented as black lines with the arrows showing the 3′ end of the strand. The red and blue circles represent s288c- and YJM789-derived SNPs, respectively. Following DSB formation, the strands are resected 5′ to 3′. One broken end invades the unbroken homolog, and DNA synthesis is primed from the 3′ invading end (shown by dotted lines). This synthesis results in the displacement of a DNA strand, allowing the displaced strand to pair with the other broken end. The resulting intermediate has two connecting Holliday junctions (HJ). If these junctions are cut by resolvases as shown by the triangles, the intermediate is resolved as a reciprocal crossover. Mismatched bases resulting from heteroduplex formation are highlighted by yellow boxes. Small arrows within the yellow boxes show the direction of mismatch repair (MMR). The red letters “A” and “B” and the blue letters “C and “D” represent the DNA sequences that flank the SNPs. Following the crossover, these flanking sequences are in the recombinant arrangement

Based on previous recombination models (Symington, et al. 2014), in Fig. 8, we show the DNA interactions likely to generate the four classes of products illustrated in Fig. 7. We assume that the recombination events are initiated by a DSB, although nick-initiated recombination cannot be ruled out. The steps for all of the events are similar. Following cleavage of the DNA, the broken ends are degraded 5′ to 3′. One end invades the unbroken centromere, forming a heteroduplex. The invaded 3′-end is used as a primer sequence for DNA synthesis resulting in displacement of the paired DNA strand from the intact duplex. The displaced strand then pairs with the other broken end (second-end capture), resulting in regions of heteroduplex flanked by two junctions. If these junctions are cleaved in different planes (as shown in Fig. 8), a reciprocal crossover will be formed. If the heteroduplexes include SNPs, mismatched bases will occur (shown by yellow highlighting). The mismatch repair system will remove one of the mismatched bases. The 4 classes identified differ in several ways: (1) whether the s288c- or YJM789-derived centromere receives the initiating DSB, (2) the extent of processing of the broken ends, (3) the amount of DNA synthesized by the invading end, and (4) whether a mismatch is repaired to generate the s288c or the YJM789 SNP allele. In addition to the interpretive diagrams of four events shown in Fig. 8, we show similar diagrams for classes and sub-classes of reciprocal recombinants in Figs. S4–S7.

This analysis leads to several interesting conclusions. First, the recombination events are initiated on the two centromeres of the dicentric chromosome with equal frequency. As shown in Fig. 8, consistent with many studies of recombination (Symington, et al. 2014), the sequence that has the initiating DSB is the recipient of information in the resulting gene conversion event. For example, as shown in Fig. 8 (I-A), when the s288c-derived centromere is broken, the resulting conversion event duplicates sequences derived from the YJM789 centromere. Of the 18 reciprocal events associated with gene conversion, 10 were initiated on the s288c GALCEN3, and 8 were initiated on the YJM789 CEN3. Second, the observation that, for most of the reciprocal recombinants, one of the interacting sequences contains exclusively s288c SNPs or YJM789 SNPs suggests that one of the junctions connecting the homologous sequences is resolved at the end of the homology (for example, Fig. 8 (I-A, II-A, and 8IV-A but not III-A). Lastly, although most heteroduplexes with more than one mismatch are repaired in the same direction (for example, Fig. 8 (II-A)), “patchy” repair is also observed (Fig. 8 (IV-A)).

### Regulation of RCO products

Depending on the choice of which strands are cut to resolve Holliday junctions, homologous recombination is predicted to give rise to CO or NCO events. In the case of dicentric chromosomes, CO events lead to the generation of linear and circular monocentric derivatives (Cook, et al. 2021). Both monocentric linear and circular derivatives are physically stable upon continued propagation on glucose. In contrast, NCO events regenerate the intact dicentric chromosome that will continue to undergo breakage and rearrangement events until monocentric derivatives arise. It has been proposed that a set of kinetochore proteins (CENP-S, X aka MHF1, MHF2) and the FACNM/Fml1/Mph1 helicase are responsible for crossover avoidance within the centromere, accounting for the high degree of gene conversion, without crossovers (Zafar, et al. 2017). The Mus81-Mms4 nuclease and Yen1 nuclease are required for optimal levels of cleavage of Holliday junctions to generate crossover products (Ho, et al. 2010).

**Fig. 9:**
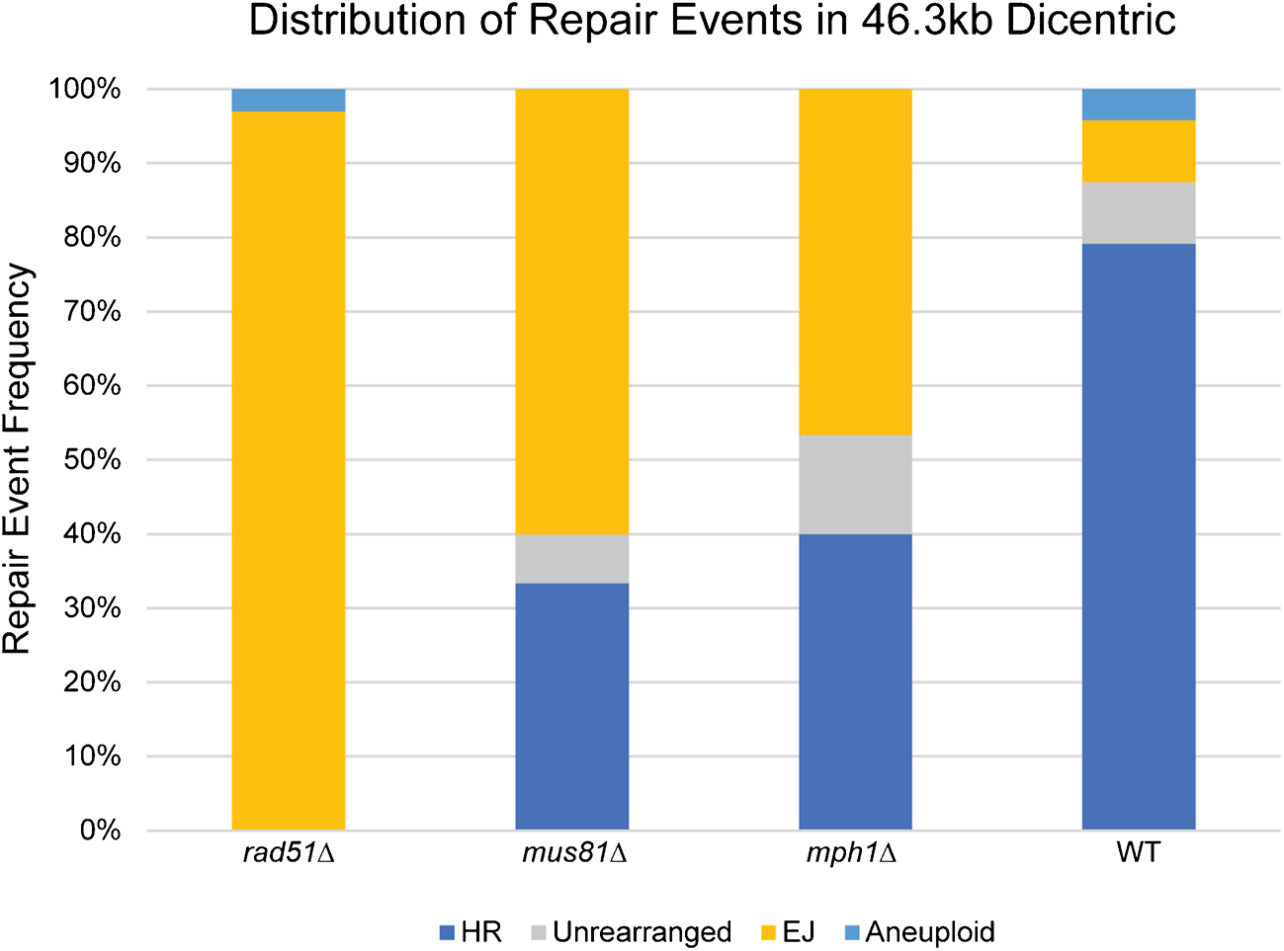
Cross-over products are reduced in *rad51Δ*, *mph1Δ*, and *mus81Δ*. Recombinant endpoint analysis of 46.3kb dicentric strains with various knockout mutations. In *rad51Δ*, the distribution of repair products is shifted almost entirely to end-joining. Since the RCO event is knocked out, any GC1-eC2 product is due to SSA, so the only survivors are EJ because the SSA event is selected against. In *mus81Δ*, HR events are less frequent than in WT (79% in WT vs 33% in *mus81Δ)*, because Mus81 promotes the RCO event required to generate the HR product. Similarly, in *mph1Δ*, HR events are reduced (79% in WT vs 40% in *mus81Δ).* See Fig. 6 legend for a detailed explanation of how each repair event was categorized. For *rad51Δ*, *n* = 30, *mus81Δ n* = 30, *mph1Δ n* = 15, WT *n* = 24 (Cook et al., 2021). Cell viability is reduced in *mus81Δ* but not in *mph1Δ.* The glu/gal viability of the 46.3kb *mus81Δ* strain is 33.7 ± 1.5% (student’s *t*-test compared to WT, *p*-value p < 0.01). The glu/gal viability of the 46.3kb *mph1Δ* strain is 55.3 ± 1.9% (student’s *t*-test compared to WT, *p*-value > 0.05). Error is the standard error of the mean, *n* = 9 for each viability assay

To examine whether Mus81 promotes the RCO events, we deleted Mus81 in cells containing the dicentric chromosome (46.3 kb) with identical centromeres. Cellular viability is reduced (25–45%, see Fig. 9 legend), but not to the extent of loss of Rad52 (6% (Cook, et al. 2021)). Analysis of single colonies after 72 h of growth on glucose reveals a decrease of about 50% (78% WT to 33% *mus81Δ*) of colonies that exhibit the RCO products (Fig. 9). Likewise, the loss of Mph1 (decreased ectopic HR events) results in a significant decrease in colonies that exhibit the RCO product (about 50%, 39% *mph1Δ* to 78% WT) (Fig. 9). The loss of Mus81 and Mph1 that influence the propensity of RCO or NCO events, respectively, decreases the accumulation of homology-based products in the population. In contrast to the results observed with *mus81Δ* and *mph1Δ*, the *rad51Δ* mutation led to a complete loss of crossovers (Fig. 9).

## Discussion

Centromeres are subject to greater mechanical tension than other genetic loci. They are integral to mitotic tension–sensing mechanisms and their proximity to microtubule plus-ends renders them vulnerable to force-induced stretching or unwinding (Lawrimore, et al. 2022). Regions of the genome that are subject to sustained tension (centromeres and rDNA) have dedicated mechanisms to ensure their physical stability (Aze, et al. 2016; Kabeche, et al. 2018; Muellner, et al. 2020). In wild-type monocentric chromosomes, these mechanisms function to reduce recombination due to fork pausing through the kinetochore protein complex and/or to suppress checkpoint activation in order to facilitate replication. We have used the conditionally functional dicentric chromosome to exert tension between two centromeres on the same DNA strand. Tension between centromeres on the same sister chromatid cannot be resolved by cohesin destruction or detangling strands. DNA breaks arise within or between the centromere in these conditions, revealing several novel aspects of centromere biology.

A homology-based pathway (SSA) is the predominant repair route between two homologous centromeres on the same chromosome (Fig. 3) (Brock, et al. 1994; Cook, et al. 2021). For homology-based repair, the centromere must be converted from an intact double-stranded structure to an intermediate with single-stranded ends. At least two scenarios might account for the production of broken ends with 3′ single-stranded “tails”. One is that random breaks occur between the centromeres followed by 5′ end resection. A second scenario is that the dicentric chromosome undergoes intra-centromeric break(s). If a DSB in one or both centromeres is able to initiate a non-reciprocal recombination event, then this scenario predicts that the kinetics of BIR or SSA would be independent of the distance between the two centromeres. As shown in Figs. 2 and S2, the appearance of the GC1-eC2 product generated by recombination or annealing of the two centromeres is comparable to 2–6 h in strains with centromeres separated between 6.5 and 46.3 kb. At later time points (48 to 72 h), genetic selection due to the essential gene (*NFS1*, 20 kb from *CEN3*) impinges on the quantity of the products in the different strains.

An independent measure of the kinetics of CEN3-GALCEN3 SSA was performed relative to *HIS4* repeats flanking the centromere (Fig. 5, (Brock et al., 1994)). The rapid kinetics of the CEN3-GALCEN3 rearrangement band (340 bp homology) relative to *HIS4* repeats (1.5 kb homology) is surprising considering that the interacting centromeres are 46.3 kb apart and the interacting *HIS4* repeats are only 5.5 kb apart. These results also suggest that centromere–centromere SSA events do not involve random breaks located between the two centromeres.

The distribution of breaks between the two centromeres has been studied in previous genetic and physical experiments. In genetic experiments, the dicentric chromosome was constructed in diploid cells using a Gal1-regulated centromere (Song, et al. 2013). In this situation, the broken dicentric is repaired via mitotic recombination with the homologous monocentric chromosome. Using strains in which single-nucleotide polymorphisms are distributed throughout the homologs, the sites of recombination between the broken chromosome and the homolog can be deduced. In this way, Song et al. (2013) demonstrated that breaks were not randomly distributed. Breaks were disproportionately localized to a 10-kb region around the endogenous or conditionally functional centromere. On chromosome III, 19/27 events were localized in the 10-kb region surrounding the centromeres, with 8/27 events localized to the intervening 20-kb. On chromosome V, 10/25 events were localized to the 10 kb proximal to the centromere, out of a 120-kb interval; 17/24 were localized to the 10 kb proximal to the centromere out of a 50-kb interval (Song, et al. 2013). On average, there was a 50% increase in the frequency of centromere-proximal breaks relative to that expected from a random distribution between the two centromeres. The use of SNPs between the two centromeres and mapping the recombination events at nucleotide resolution (Figs. 6–8) extend the Song et al. (2013) study and reveal the centromere DNA element II (CDEII) as the primary site of breakage.

Physical analysis of dicentric chromosomes revealed the 25–30-kb region around the centromere to be the predominant site of breakage (Lopez, et al. 2015). In this study, a conditional centromere was introduced into chromosome VII. Following the activation of the conditional centromere, chromosome fragments were separated by size on pulsed-field gel electrophoresis and hybridized to radio-labeled probes with homology to the left or right arm of the chromosome. Two predominant bands revealing breakage at either the endogenous or conditional centromere location were evident (Lopez, et al. 2015). Thus, both genetic and physical studies are indicative of the centromere as the preferred site of breakage following activation of a dicentric chromosome.

In this study, we used centromeres with several single-nucleotide polymorphisms between them (Figs. 6–8). Strains with these two centromeres in chromosome III are viable, as are strains with identical CEN3 sequences. However, in the SNP dicentric, the repair products shifted from predominantly homologous recombination to 69% NHEJ, 7% HR, and 24% cells with both centromeres intact (Fig. 6). The latter events are likely monocentric derivatives that arise from homologous recombination between Ty elements at 84 and 149 kb (Hill, et al. 1989; Surosky, et al. 1985). The reduced frequency of the HR events in the SNP strain (7% vs 78%) is consistent with the degree of mismatch between the two centromeres. A few percent-mismatched bases significantly reduce the frequency of homologous recombination between ectopic repeats (Datta, et al. 1997). Examination of the products in these cells reveals that recombination was initiated with about equal frequency within the conserved centromere DNA elements CDEII and CDEIII of either the GALCEN3 or CEN3. The conversion tracts range from about 50 bp to the full length of the homology between the two centromeres (340 bp). This data provides unequivocal evidence for breaks within the centromere on the dicentric chromosome.

**Fig. 10:**
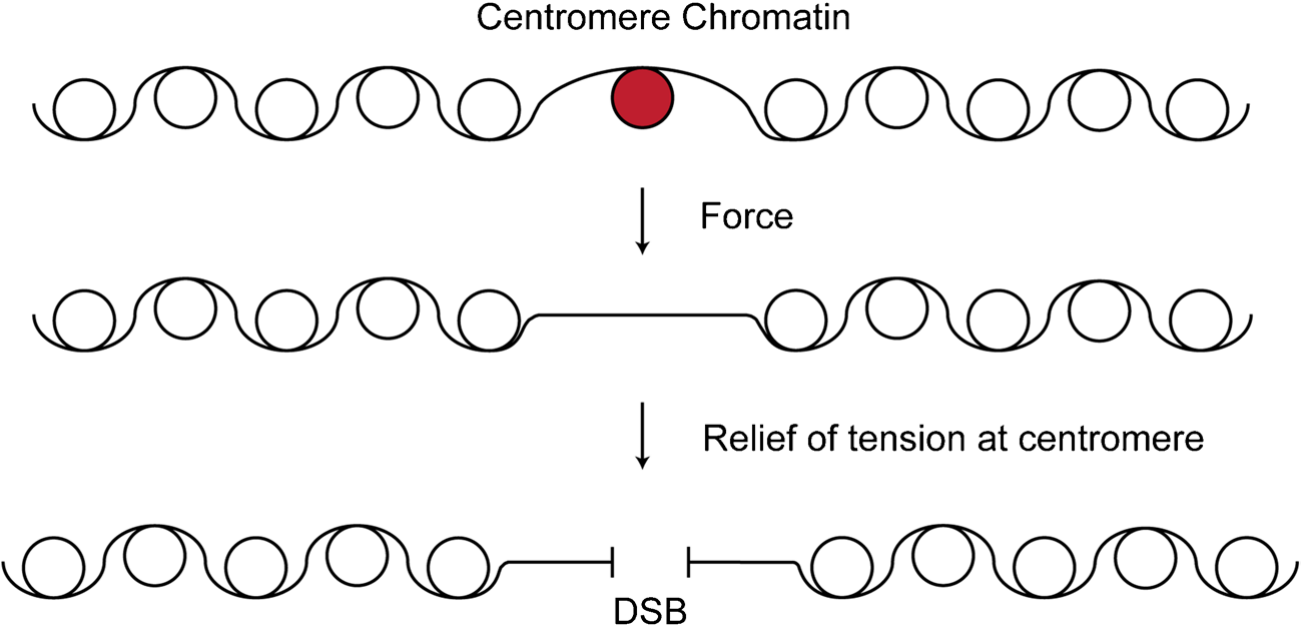
Force-induced nucleosome eviction converts the centromere into a chromosome-fragile site. We propose that activation of the dicentric chromosome results in the exposure of protein-free centromere DNA through microtubule-based pulling forces. Force exertion will increase the frequency of nucleosome release (Yan et al., 2007). Alternatively, microtubule-based forces may exacerbate the progression of the replication fork through the centromere, increasing the propensity for fork stalling. Tension is relieved upon the formation of a DSB within the centromere. Throughout the normal course of cell cycle progression, the centromere is protected from nucleolytic attack due to centromere-specific histone H3 binding (*CSE4*) as well as kinetochore proteins (Bloom et al., 1982; Furuyama et al., 2007). Upon exertion of mechanical force on the centromere through the dicentric configuration where centromeres on the same sister chromatid attach to opposite spindle poles, the dissociation of centromere proteins is increased, rendering the centromere susceptible to damage

The cellular response to activation of the dicentric chromosome is a mid-anaphase pause (Yang, et al. 1997), indicative of a mechanism that can delay cells with DNA damage prior to mitotic exit and cytokinesis (Tinker-Kulberg, et al. 1999). At this stage of the cell cycle, the 30 sister monocentric chromosomes have segregated to their respective poles, while the dicentric lags behind and can be observed straddled between the two poles (see Fig. 3C in (Joglekar, et al. 2006)). If kinetochore-microtubule attachments persist, recoil of the dicentric chromosome will pull the spindle poles toward one another (Lopez, et al. 2015). The spindle collapses to the neck of the budded cell, where centromeres are preferentially severed due to their proximity to the bud neck (Guérin, et al. 2019; Lopez, et al. 2015). Physical shearing of the centromere through growth of a new cell wall (100–200 nm) is likely to delete much more than the centromere and adjacent nucleosomes (Lopez, et al. 2015). To account for the high efficiency of DNA repair observed following dicentric chromosome activation, damage at or between the centromeres is likely to be mitigated. Exertion of force along the double helix promotes the dissociation of DNA-binding proteins, including canonical histones and the centromere-specific histone H3 variant, CENP-A (Fig. 10) (Dahlke, et al. 2019; Kim, et al. 2016). The increased off-rate of CENP-A will increase the exposure of the core centromere containing the A+T-rich CDEII and CDEIII to enzymatic attack. In this way, forces render the centromere as a potential target for DNA damage and subsequent repair.

## Materials and Methods

### Strain construction

Direct dicentric strain construction is described in Cook et al. (2021) (Table 1). Inverted dicentric strains were constructed using the same method and screened using the same primers as the direct dicentrics. The 6.5-kb inverted dicentric was constructed using Ldb16 reverse top: GCACATACACTTATGTGGTTCACCGTGCCGCTGCTGTGTTTATCTGTTGCTCGACATGTGCTGCATTAGGAAGCAGCCCAGTAGTA and Ldb16 reverse bottom: TTTCTCACTATAAAAAAAGAAGAAATTACTTTAAATTGTTTGTCTATTCCAACATAATCATTAGAGATCCAGTTCGATGTAACC. The 9.8-kb inverted dicentric was constructed using Ilv6 reverse top: CTTAGAGAAGCCACCAAGGTATTGTGTCTTTAAGAAGCAGCCCAGTAGTA and Ilv6 reverse bottom: GTACGTTTGTACGAGGTGACGCGTTACTAAACTATTTTTTTCTTTTGGTTTTCTGCTTTCCAGATCCAGTTCGATGTAACC. The 12.3-kb inverted dicentric was constructed using Gbp2 reverse top: ATCGCTGGAAGTGGTGCTCTTTTACAGGGATTAGGAAGCAGCCCAGTAGTA and Gbp2 reverse bottom: GATAACGTATAAATAATAAGGAAGCGGGCGGGTTATAAATAACTTTAATAGTTATATTTATAGATCCAGTTCGATGTAACC. The 18.2-kb dicentric was constructed using Dcc1 reverse top: CTCCTAGAGATTTCGATCACCATCGTGGTGCTCTTTGTCATACGCATAGAATTGACAAAACATTAGGAAGCAGCCCAGTAGTA and Dcc1 reverse bottom: ACCCTAGGTCTTGGCAACTGGCAATCGCTAACATGACCTAATTTATAGCTTAGGGTTCTAGATCCAGTTCGATGTAACC. The 46.3-kb inverted dicentric was constructed using GalCenHyg-His4-indirect top: AGTTTCACCCTTGATCCAGATTTCATTCCTAGAACGAGAATAATAAACGCCACGACCCAACATTAGGAAGCAGCCCAGTAGTA and GalCenHyg-His4-indirect bottom: AGTGCTTGGTGAAGTACGTACAGACCGTCCTGACGGTTTATATACCACCCTAGTTGTCGACAGATCCAGTTCGATGTAACC.

**Table 1:** Strain table

J1781D	MATa ade1 met14 ura3-52 leu2-3,112 his3-11,15
DCY1232.1	J1781D GALCEN3-HB::ldb16
SLY2.1	J1781D GALCEN3-HB::ilv6
SLY6.1	J1781D GALCEN3-HB::gbp2
SLY1.1	J1781D GALCEN3-HB::dcc1
DCY1214.1	J1781D GALCEN3-HB::his4
SLY5.1	J1781D InvertedGALCEN3-HB::ldb16
SLY4.1	J1781D InvertedGALCEN3-HB::ilv6
SLY7.1	J1781D InvertedGALCEN3-HB::gbp2
SLY3.1	J1781D InvertedGALCEN3-HB::dcc1
SLY15.1	J1781D InvertedGALCEN3-HB::his4
DCY1408.1	J1781D GALCEN3-ura::his4 rad1::kan
DCY1385.1	J1781D GALCEN3-ura::his4 rad51::HIS3
DCY1430.1	J1781D GALCEN3-HB::ilv6 rad51::HIS3
DCY1238.1	J1781D GALCEN3-HB::ldb16 rad52::LEU2
SLY8.1	J1781D GALCEN3-HB::ilv6 rad52::LEU2
SLY11.1	J1781D GALCEN3-HB::gbp2 rad52::LEU2
SLY9.1	J1781D GALCEN3-HB::dcc1 rad52::LEU2
DCY1227.1	J1781D GALCEN3-HB::his4 rad52::LEU2
J178#4	J1781D GALCEN3-ura::his 4 pR285-GALCEN-I
J178#7	J1781D GALCEN3-ura::his4 pR285-GALCEN-D
DCY1393.3	J1781D GALCEN3-HB::his4 mus81::nat
DCY1437.1	J1781D GALCEN3-HB::his4 mph1::nat
DCY1391.1	J1781D GALCEN3-HB::his4 tof1::nat
DCY1131.1	J1781D GALCEN3-HB::his4 mrc1::nat
DCY1202.1	J1781D GALCEN3-ura::his4 asf1::nat
DCY1414.1	J1781D GALCEN3-HB::his4 csm3::nat
DCY1397.1	J1781D GALCEN3-HB::his4 rtt109::nat
1214-9	J1781D GALCEN3-HB::his4 (original CEN3 replaced with CEN3 from YJM789)
1214-21	J1781D GALCEN3-HB::his4 (original CEN3 replaced with CEN3 from YJM789)

Dicentric mutants were constructed by generating a knockout PCR product with various markers using the pFA6 plasmid series. Transformants were screened with flanking primers to confirm gene deletion. Strain construction for Rad52 and Mrc1 mutants is described in Cook et al. (2021). Rad1 was knocked out using rad1kanC: GAGAGAGCACAGGTGTACTGGAGGGTTCAGGACGTTGGTAGAGCATTTGCCAGCTGAAGCTTCGTACGC and rad1kanD: GATGTCTAACTTATAACATATACGGTTGGAAGTCACCAAATGAATATTGTTTAGGCCACTAGTCGATCTG and screened with rad1C:TTGTCTGCGTGGCGCATAGG and rad1D:GTGAATAAACTATGAACGCGAA. Rad51 was knocked out using rad51 up: TGAGTGTAGCGACAAAGAGCAGACGTAGTTATTTGTTAAAGGCCTACTAATTTGTTATCGTCATCGGATCCCCGGGTTAATTAA and rad51 dn: CGCAACCTAAGAAAAAGAGGAGAATTGAAAGTAAACCTGTGTAAATAAATAGAGACAAGAGACCAGAATTCGAGCTCGTTTAAAC and screened with Rad51 chk up: TTTGTTTACAGTACGCGTGG and Rad51 chk dn: TTTCCATCCACTGTCTTAGA. Mus81 was knocked out using Mus81 top: GCATCAACATTGGCGTAAACAAAGTTTCAAAGGATTGATACGAACACACATTCCTAGCATGAAAGCCGGATCCCCGGGTTAATTAA and Mus81 bot: TTGTCAAGTGGCATCATAATGCATTGGGGCGGCTTTCAGATATGCTTCTGGTATATTTGTCCGTGAGAATTCGAGCTCGTTTAAA and screened with Mus81 chk up: CACGTATACTTACCATCTATAGTGTTA and Mus81 chk dn: TTCAATGACATATCTGAGCACTATTA. Mph1 was knocked out with Mph1 KO up: AGAGGTGCCCTATGCTCTATCACGGAGCTAAGATATTGTGATTCAAGATATAGTAGCTCACTTCCGGATCCCCGGGTTAATTAA and Mph1 KO dn: CATGACAGAAAGGTTTTGCATTGGTAGGCGTGGAAGATTACAGATTGTACTCGTCGTTGGCTCGAATTCGAGCTCGTTTAAAC and screened with Mph1 chk up: TAGCTTACTGTGCTCACAGAAAGACATAAACCG and Mph1 chk dn: TAGGCCTGGTTGGAGAGCGAAAGTTGAGATT. Tof1 insertion was knocked out using Tof1 KO up: CACATATGATAATACCATCTAGCTTGTGGGGTTTAGTGTATCTTTAATATAGGAGGGCGCACACTCGGATCCCCGGGTTAATTAA and Tof1 KO dn: AATTACACGTATTAAAGGGATTAATTACTACATATTCATTCTCAATCATCACTATCACCTTGGCTCGAATTCGAGCTCGTTTAAAC and screened with Tof1 chk up: TTAGGAAGCTGTTCTGTGTTACTAT and Tof1 chk dn: TGATGACAATTTCGATGATGCCCAGGA. Asf1 was knocked out using asf1 5′ KO: TCGAAAGTGTAACAGCGTACTCTCCCTACCATCCAATTGAAACATAAGATATAGAAAAGCGGATCCCCGGGTTAATTAA and asf1 3′ KO: CATTTTATAAAGTGTACCTCTCTTGCAGGTACCATTAATCTTATAACCCATAAATTCGAATTCGAGCTCGTTTAAAC and screened with Asf1 5′ chk: GAGAGAGCTGTTCTACAAAGAACTT and Asf1 3’ chk: TGTCATACTGACGTATCTCACTTTG. Csm3 was knocked out using Csm3 up: CTGACGCGTAACAAGATCAATGATATACTGGATTAAAATGCCATGAAAACGTGAACGGATCCCCGGGTTAATTAA and Csm3 dn: CCTATATGTATAGATGCCCACACGCACGTTTGGATTATTACCTTCAATGACATTGCGAATTCGAGCTCGTTTAAAC and screened with Csm3 chk up: CATGGTAAGGAAAAATGCCGGGTAAT and Csm3 chk dn: CTCGAACCAGGCTCTTTCTACAAGC. Rtt109 was knocked out using Rtt109 up: CTACAGTTTGTCAATAGAGTTGTCCAGTAGAGTTAAAAGGTCAATTCAACCGGTCTTCAATAAGACCGGATCCCCGGGTTAATTAA and Rtt109 dn: TACAAACATGCATTTTCTAAGATCGATGCTACATACGTGTACTAAATAATAAATATCAATATGTAGAATTCGAGCTCGTTTAAAC and screened with Rtt109 chk up: ATGGACGCCATAACGCATT and Rtt109 chk dn: GAGGACCTATCATTACCTAG.

We constructed two nearly isogenic dicentric strains (DCY1214-9 and DCY1214-21) in which the GAL CEN sequence was derived from s288c, and the CEN3 sequence at its native location was derived from the haploid strain YJM789. In the first step of this construction, a 3.3-kb DNA fragment containing containing the KIURA3 and kanMX4 genes was generated by PCR amplification using the pCORE template (Storici, et al. 2001) and the primers: cen3-core-F cen3-core-F (5′-TTT CAT CAC CCA CAT TAT AGT ACA AAC CTA CTG GTG TAA CCA TTA TCA TAT TCA TGA CTT GAG CTC GTT TTC GAC ACT GG) and cen3-core-R (5′-TGA TAT GAA ACT ATT TAA CGT GAT TTT TTC CTC AAT TTA TCG TGA AAG ATT TTT AAC TAC TCC TTA CCA TTA AGT TGA TC). This fragment was used to transform the dicentric yeast strain DCY1214. Ura+ transformants were selected on a synthetic complete medium without uracil (Rose, et al. 1990) supplemented with 2% galactose (and containing no glucose) (SCGal-URA). The integration of this cassette 546 bp from CEN3 (between SGD coordinates 115,046 and 115,047) was confirmed by PCR with the primer pairs: (1) URA3.2 (5′-AGA CGA CAA AGG CGA TGC AT-3′) (Storici, et al. 2001) plus cen3-regR (5′-TAT ATA CAA TGT TGT GAC AG-3′) and (2) kanFW (5′-CCT CGA CAT CAT CTG CCC-3′) (Güldener, et al. 1996) plus cen3-verF2 (5′-TCC GCT TAT AGT ACA GTA CC-3′). In the second step of the construction, a 1475-bp fragment containing the YJM789 CEN3 was generated by PCR amplification of genomic DNA from YJM789 using the primers: cen3-regF (5′-ACT TAT TAC AGA TAG TGT AC-3′) and cen3-regR (described above). This PCR product was used to replace the KIURA3/kanMX4 cassette of the Ura+ DCY1214 derivative with the YJM789 CEN3 and flanking sequences. This replacement was selected using medium containing 5-fluoro-orotate (5-FOA) and uracil. The 5-FOA-resistant derivatives were screened by replica plating to identify those that lost the kanMX. The 340-bp CEN3-containing region was sequenced to confirm the presence of the YJM789 CEN3; the PCR fragment that was sequenced was generated using the primers cen3-regF and cen3-regR. For several isolates, we also sequenced the flanking region. Two isolates were chosen for our further studies. In the isolate DCY1214-9, the 1042-bp YJM789-derived DNA fragment replaced the 1044-bp CEN3-containing s288c DNA fragment, corresponding to Saccharomyces Genome Database coordinates 114,169 to 115,212. In the isolate DCY1214-21, 1064 bp of YJM789 sequences replaced 1066 bp of s288c sequences, corresponding to SGD coordinates 113,921 to 114,986.

Analysis of dicentric strains was performed with robes for parental and repair products eC1: TCAATAGCTTGCAGCGTAGCTAA, eC2: GGGTGGGAAACTGAAGAAATC, GC1: TCGACTACGCGATCATGGCG, GC2: CACGATGCGTCCGGCGTAGA, his4popout seq1: TCCGAATCACAGTCAGTAGAGATTTG, and his4 popout seq 4: CAGCGGTAATCAACAGTCGATGGACCAG.

### Yeast media

Cells were grown on liquid and solid Yeast Peptone Dextrose (YPD) and Yeast Peptone Galactose (YPG). All cultures were grown at 24° in an orbital shaker.

### Viability

Five milliliter overnight cultures were grown in YPG, dilutions were made in sterile water which were then plated with glass beads on YPG and YPD plates. Single colonies were counted after 2 weeks of incubation at 24°. Viability is calculated by dividing the number of colonies on glucose by the number of colonies on galactose plates.

### Time course

Five milliliter overnight cultures were grown in YPG. Cultures were diluted to an OD660 of 0.15 in 25 mL YPG and grown for 1.5 h. The cultures were pelleted for 5 min at 3000 rpm, resuspended in YPD, and allowed to grow for a total of 72 h. The 1.5 mL aliquots were taken at hours 2, 4, 6, and 24, then 1 mL of the culture was reinoculated into a fresh 25 mL YPD culture. An aliquot was taken at 48 h and another 1 mL reinoculation occurred. The final aliquot was taken at 72 h. Also at 72 h, cells were diluted as described above and plated on YPD and YPG. Single colonies from the YPD plates were picked for further analysis as described in the “Endpoint analysis” section. The time courses described in Figs. 2, 3, and 4 were repeated independently three times and analyzed separately.

### Time course polymerase chain reaction (PCR) and quantitation

Genomic DNA from each time point was prepared using a phenol-chloroform extraction and diluted to 10 ng/μL. PCR was set up in triplicate using GoTAQ Green Mastermix (Promega, Madison, WI) in 25 μL reactions and 1 μL of diluted and equalized DNA template. In Fig. S1, 5 μL of PCR product was run on a gel, stained with EtBr, and imaged on a ChemiDoc imager (Biorad, Hercules, CA). DNA quantitation was performed using a qBit Fluorimeter (Invitrogen, Waltham, MA). Using the 1× DS DNA assay kit, DNA concentration was measured for each product. Values were averaged and plotted in line charts and/or bar charts. A no-product-control PCR reaction (included on bar charts but not line charts) was performed for both primer sets (GC1-eC2, eC1-GC2) in triplicate with DNA from a WT monocentric yeast strain (J1781D) that could not produce either repair product. This no-product control reflects the concentration of the input DNA in the PCR reaction only. A standard PCR protocol was used throughout 98° C for 2 min followed by 30 cycles of 95° C for 1 min, 52° C for 30 s, 68° C for 3 min, then 68° C for 5 min and held at 4° C.

### Gel intensity quantitation for His4 rearrangement (Fig. 5)

Time courses were completed as described above and DNA was diluted to 1 ng/μL, 1 μL of which was used as a template for PCR as described above. PCR was set up in duplicate using primers GC1-eC2, eC1-GC2, and 1-4 for the HIS4 rearrangement fragment. A total of 5 μL of each PCR was combined twice and run in two wells of a large 1% agarose gel. The gel was stained with 0.5 μg/mL EtBr and imaged on a ChemiDoc imager (Biorad, Hercules, CA). The box-in-box method (Hoffman, et al. 2001) was used to quantify the integrated intensity of each gel band in Metamorph. Each plotted intensity is an average of the two replicates from the same gel. Gels run throughout were run at constant amperage (200 mA) and used 5 μL of Generuler 1 kb Plus DNA Ladder (Thermo Scientific, Waltham, MA).

### Endpoint analysis

After the completion of a 72-h time course plating, single colonies were picked into 2 mL YPD cultures and allowed to grow overnight. Genomic DNA was prepared as described above and 1 μL was used as a template in a standard 25 μL PCR reaction. For each single colony, the following four reactions were run: parental products CEN3 (eC1-eC2), GALCEN3 (GC1-GC2), and recombinant products (eC1-GC2) and (GC1-eC2). A total of 5 μL of PCR product was run on a gel and each colony was scored for the presence or absence of a WT-size parental product or recombinant product and assigned a repair category.

### Sequencing

Recombinant products were sequenced via Genewiz (Azenta) Sanger sequencing. Products were prepared for sequencing using the GeneJET PCR Purification Kit (Thermo Scientific). Sequence files were analyzed using SnapGene and Benchling.

### Supplementary information

ESM 1 (DOCX 2213 kb)

## Data Availability

All relevant data are within the manuscript and its supporting information files.

## References

[CR1] Akiyoshi B, Sarangapani KK, Powers AF, Nelson CR, Reichow SL, Arellano-Santoyo H, Gonen T, Ranish JA, Asbury CL, Biggins S (2010). Tension directly stabilizes reconstituted kinetochore-microtubule attachments. Nature.

[CR2] Altemose N, Logsdon GA, Bzikadze AV, Sidhwani P, Langley SA, Caldas GV, Hoyt SJ, Uralsky L, Ryabov FD, Shew CJ, Sauria MEG, Borchers M, Gershman A, Mikheenko A, Shepelev VA, Dvorkina T, Kunyavskaya O, Vollger MR, Rhie A, McCartney AM, Asri M, Lorig-Roach R, Shafin K, Lucas JK, Aganezov S, Olson D, de Lima LG, Potapova T, Hartley GA, Haukness M, Kerpedjiev P, Gusev F, Tigyi K, Brooks S, Young A, Nurk S, Koren S, Salama SR, Paten B, Rogaev EI, Streets A, Karpen GH, Dernburg AF, Sullivan BA, Straight AF, Wheeler TJ, Gerton JL, Eichler EE, Phillippy AM, Timp W, Dennis MY, O'Neill RJ, Zook JM, Schatz MC, Pevzner PA, Diekhans M, Langley CH, Alexandrov IA, Miga KH (2022). Complete genomic and epigenetic maps of human centromeres. Science.

[CR3] Aze A, Sannino V, Soffientini P, Bachi A, Costanzo V (2016). Centromeric DNA replication reconstitution reveals DNA loops and ATR checkpoint suppression. Nat Cell Biol.

[CR4] Bhattacharjee S, Osman F, Feeney L, Lorenz A, Bryer C, Whitby MC (2013). MHF1-2/CENP-S-X performs distinct roles in centromere metabolism and genetic recombination. Open Biol.

[CR5] Bloom K, Costanzo V (2017). Centromere structure and function. Prog Mol Subcell Biol.

[CR6] Bloom KS, Carbon J (1982). Yeast centromere DNA is in a unique and highly ordered structure in chromosomes and small circular minichromosomes. Cell.

[CR7] Branzei D, Foiani M (2010). Maintaining genome stability at the replication fork. Nat Rev Mol Cell Biol.

[CR8] Brock JA, Bloom K (1994). A chromosome breakage assay to monitor mitotic forces in budding yeast. J Cell Sci.

[CR9] Cha RS, Kleckner N (2002). ATR homolog Mec1 promotes fork progression, thus averting breaks in replication slow zones. Science.

[CR10] Chacon JM, Mukherjee S, Schuster BM, Clarke DJ, Gardner MK (2014). Pericentromere tension is self-regulated by spindle structure in metaphase. J Cell Biol.

[CR11] Cook D, Long S, Stanton J, Cusick P, Lawrimore C, Yeh E, Grant S, Bloom K (2021). Behavior of dicentric chromosomes in budding yeast. PLoS Genet.

[CR12] Dahlke K, Zhao J, Sing CE, Banigan EJ (2019). Force-dependent facilitated dissociation can generate protein-DNA catch bonds. Biophys J.

[CR13] Datta A, Hendrix M, Lipsitch M, Jinks-Robertson S (1997). Dual roles for DNA sequence identity and the mismatch repair system in the regulation of mitotic crossing-over in yeast. Proc Natl Acad Sci U S A.

[CR14] Drinnenberg IA, Henikoff S, Malik HS (2016). Evolutionary turnover of kinetochore proteins: a ship of Theseus?. Trends Cell Biol.

[CR15] Furuyama S, Biggins S (2007). Centromere identity is specified by a single centromeric nucleosome in budding yeast. Proc Natl Acad Sci U S A.

[CR16] Greenfeder SA, Newlon CS (1992). Replication forks pause at yeast centromeres. Mol Cell Biol.

[CR17] Guérin TM, Béneut C, Barinova N, López V, Lazar-Stefanita L, Deshayes A, Thierry A, Koszul R, Dubrana K, Marcand S (2019). Condensin-mediated chromosome folding and internal telomeres drive dicentric severing by cytokinesis. Mol Cell.

[CR18] Güldener U, Heck S, Fielder T, Beinhauer J, Hegemann JH (1996). A new efficient gene disruption cassette for repeated use in budding yeast. Nucleic Acids Res.

[CR19] Hashash N, Johnson AL, Cha RS (2012). Topoisomerase II- and condensin-dependent breakage of MEC1ATR-sensitive fragile sites occurs independently of spindle tension, anaphase, or cytokinesis. PLoS Genet.

[CR20] Henikoff JG, Thakur J, Kasinathan S, Henikoff S (2015). A unique chromatin complex occupies young α-satellite arrays of human centromeres. Sci Adv.

[CR21] Hill A, Bloom K (1989). Acquisition and processing of a conditional dicentric chromosome in *Saccharomyces cerevisiae*. Mol Cell Biol.

[CR22] Ho CK, Mazón G, Lam AF, Symington LS (2010). Mus81 and Yen1 promote reciprocal exchange during mitotic recombination to maintain genome integrity in budding yeast. Mol Cell.

[CR23] Hodgson B, Calzada A, Labib K (2007). Mrc1 and Tof1 regulate DNA replication forks in different ways during normal S phase. Mol Biol Cell.

[CR24] Hoffman DB, Pearson CG, Yen TJ, Howell BJ, Salmon ED (2001). Microtubule-dependent changes in assembly of microtubule motor proteins and mitotic spindle checkpoint proteins at PtK1 kinetochores. Mol Biol Cell.

[CR25] Joglekar AP, Bouck DC, Molk JN, Bloom KS, Salmon ED (2006). Molecular architecture of a kinetochore-microtubule attachment site. Nat Cell Biol.

[CR26] Kabeche L, Nguyen HD, Buisson R, Zou L (2018). A mitosis-specific and R loop-driven ATR pathway promotes faithful chromosome segregation. Science.

[CR27] Kim SH, Vlijm R, van der Torre J, Dalal Y, Dekker C (2016). CENP-A and H3 nucleosomes display a similar stability to force-mediated disassembly. PloS One.

[CR28] Kobayashi T, Sasaki M (2017) Ribosomal DNA stability is supported by many ‘buffer genes’-introduction to the yeast rDNA stability database. FEMS Yeast Res 17. 10.1093/femsyr/fox001 10.1093/femsyr/fox001 28087673

[CR29] Kursel LE, Malik HS (2017). Recurrent gene duplication leads to diverse repertoires of centromeric histones in *Drosophila* species. Mol Biol Evol.

[CR30] Lawrimore J, Bloom K (2022) Shaping centromeres to resist mitotic spindle forces. J Cell Sci 135. 10.1242/jcs.259532 10.1242/jcs.259532 PMC8919341 35179192

[CR31] Liebman SW, Symington LS, Petes TD (1988). Mitotic recombination within the centromere of a yeast chromosome. Science.

[CR32] Liu L, Malkova A (2022). Break-induced replication: unraveling each step. Trends Genet.

[CR33] Lopes M, Foiani M, Sogo JM (2006). Multiple mechanisms control chromosome integrity after replication fork uncoupling and restart at irreparable UV lesions. Mol Cell.

[CR34] Lopez V, Barinova N, Onishi M, Pobiega S, Pringle JR, Dubrana K, Marcand S (2015). Cytokinesis breaks dicentric chromosomes preferentially at pericentromeric regions and telomere fusions. Genes Dev.

[CR35] McFarlane RJ, Humphrey TC (2010). A role for recombination in centromere function. Trends in genetics: TIG.

[CR36] Muellner J, Schmidt KH (2020) Yeast genome maintenance by the multifunctional PIF1 DNA helicase family. Genes 11. 10.3390/genes11020224 10.3390/genes11020224 PMC7073672 32093266

[CR37] Nurk S, Koren S, Rhie A, Rautiainen M, Bzikadze AV, Mikheenko A, Vollger MR, Altemose N, Uralsky L, Gershman A, Aganezov S (2022). The complete sequence of a human genome. Science.

[CR38] Padmanabhan S, Thakur J, Siddharthan R, Sanyal K (2008). Rapid evolution of Cse4p-rich centromeric DNA sequences in closely related pathogenic yeasts, *Candida albicans* and *Candida dubliniensis*. Proc Natl Acad Sci U S A.

[CR39] Prakash R, Satory D, Dray E, Papusha A, Scheller J, Kramer W, Krejci L, Klein H, Haber JE, Sung P, Ira G (2009). Yeast Mph1 helicase dissociates Rad51-made D-loops: implications for crossover control in mitotic recombination. Genes Dev.

[CR40] Ramakrishnan S, Kockler Z, Evans R, Downing BD, Malkova A (2018). Single-strand annealing between inverted DNA repeats: pathway choice, participating proteins, and genome destabilizing consequences. PLoS Genet.

[CR41] Romeo F, Falbo L, Costanzo V (2016). Replication, checkpoint suppression and structure of centromeric DNA. Nucleus.

[CR42] Rose MD, Winston FM, Heiter P (1990). Methods in yeast genetics: a laboratory course manual.

[CR43] Shi J, Wolf SE, Burke JM, Presting GG, Ross-Ibarra J, Dawe RK (2010). Widespread gene conversion in centromere cores. PLoS Biol.

[CR44] Song W, Gawel M, Dominska M, Greenwell PW, Hazkani-Covo E, Bloom K, Petes TD (2013). Nonrandom distribution of interhomolog recombination events induced by breakage of a dicentric chromosome in Saccharomyces cerevisiae. Genetics.

[CR45] Storici F, Lewis LK, Resnick MA (2001). In vivo site-directed mutagenesis using oligonucleotides. Nat Biotechnol.

[CR46] Surosky RT, Tye BK (1985). Resolution of dicentric chromosomes by Ty-mediated recombination in yeast. Genetics.

[CR47] Symington LS, Petes TD (1988). Meiotic recombination within the centromere of a yeast chromosome. Cell.

[CR48] Symington LS, Rothstein R, Lisby M (2014). Mechanisms and regulation of mitotic recombination in *Saccharomyces cerevisiae*. Genetics.

[CR49] Talbert PB, Henikoff S (2010). Centromeres convert but don't cross. PLoS Biol.

[CR50] Thrower DA, Stemple J, Yeh E, Bloom K (2003). Nuclear oscillations and nuclear filament formation accompany single-strand annealing repair of a dicentric chromosome in *Saccharomyces cerevisiae*. J Cell Sci.

[CR51] Tinker-Kulberg RL, Morgan DO (1999). Pds1 and Esp1 control both anaphase and mitotic exit in normal cells and after DNA damage. Genes Dev.

[CR52] Wei W, McCusker JH, Hyman RW, Jones T, Ning Y, Cao Z, Gu Z, Bruno D, Miranda M, Nguyen M, Wilhelmy J, Komp C, Tamse R, Wang X, Jia P, Luedi P, Oefner PJ, David L, Dietrich FS, Li Y, Davis RW, Steinmetz LM (2007). Genome sequencing and comparative analysis of Saccharomyces cerevisiae strain YJM789. Proc Natl Acad Sci.

[CR53] Yan J, Maresca TJ, Skoko D, Adams CD, Xiao B, Christensen MO, Heald R, Marko JF (2007). Micromanipulation studies of chromatin fibers in *Xenopus* egg extracts reveal ATP-dependent chromatin assembly dynamics. Mol Biol Cell.

[CR54] Yan Z, Xue C, Kumar S, Crickard JB, Yu Y, Wang W, Pham N, Li Y, Niu H, Sung P, Greene EC, Ira G (2019). Rad52 restrains resection at DNA double-strand break ends in yeast. Mol Cell.

[CR55] Yang SS, Yeh E, Salmon ED, Bloom K (1997). Identification of a mid-anaphase checkpoint in budding yeast. J Cell Biol.

[CR56] Zafar F, Okita AK, Onaka AT, Su J, Katahira Y, Nakayama JI, Takahashi TS, Masukata H, Nakagawa T (2017). Regulation of mitotic recombination between DNA repeats in centromeres. Nucleic Acids Res.

[CR57] Zhang H, Freudenreich CH (2007). An AT-rich sequence in human common fragile site FRA16D causes fork stalling and chromosome breakage in *S. cerevisiae*. Mol Cell.

