# Supplementary material for: Dicentric chromosomes are resolved through breakage and repair at their centromeres
Source: Chromosoma. 2024 Jan 2;133(2):117–34. doi: 10.1007/s00412-023-00814-6 (PMC11180013; doi:10.1007/s00412-023-00814-6)
Supplement: Supplementary file 1 — (DOCX 2213 kb) [file 412_2023_814_MOESM1_ESM.docx]

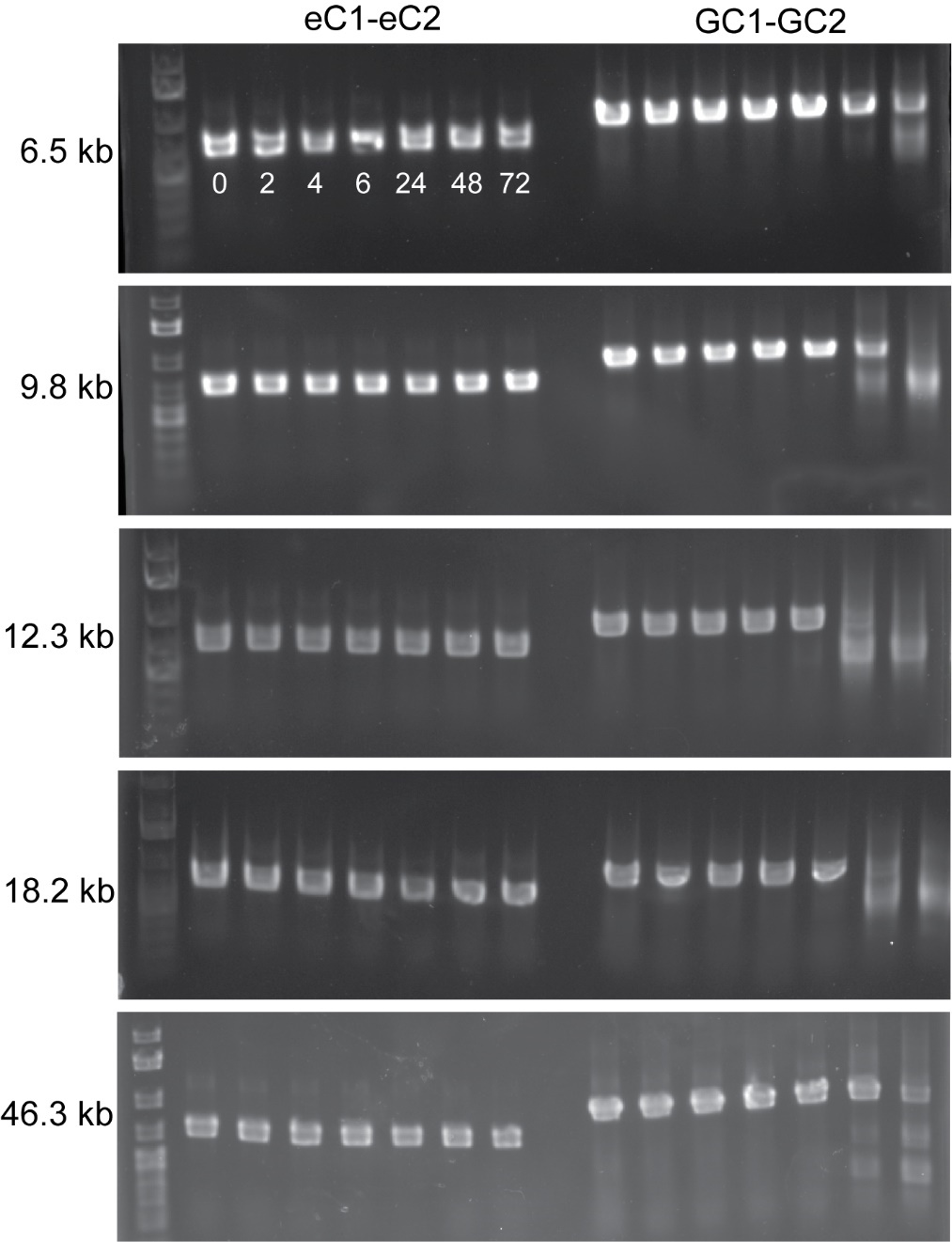


Figure S1. **GALCEN3 accumulates small deletions over time.** Gels of parental products CEN3 (eC1-eC2) and GALCEN3 (GC1-GC2) in strains with GALCEN3 inserted 6.5, 9.8, 12.3, 18.2, and 46.3 kb away from the endogenous CEN3, after switching carbon source from galactose to glucose (GALCEN3 activation) and growing for 72 hours. There is a slight decrease in intensity of the CEN3 band and a more marked decrease of the GALCEN3 band, the timing of which corresponds to the onset of small deletions which appear as smaller GC1-GC2 bands on the gel.


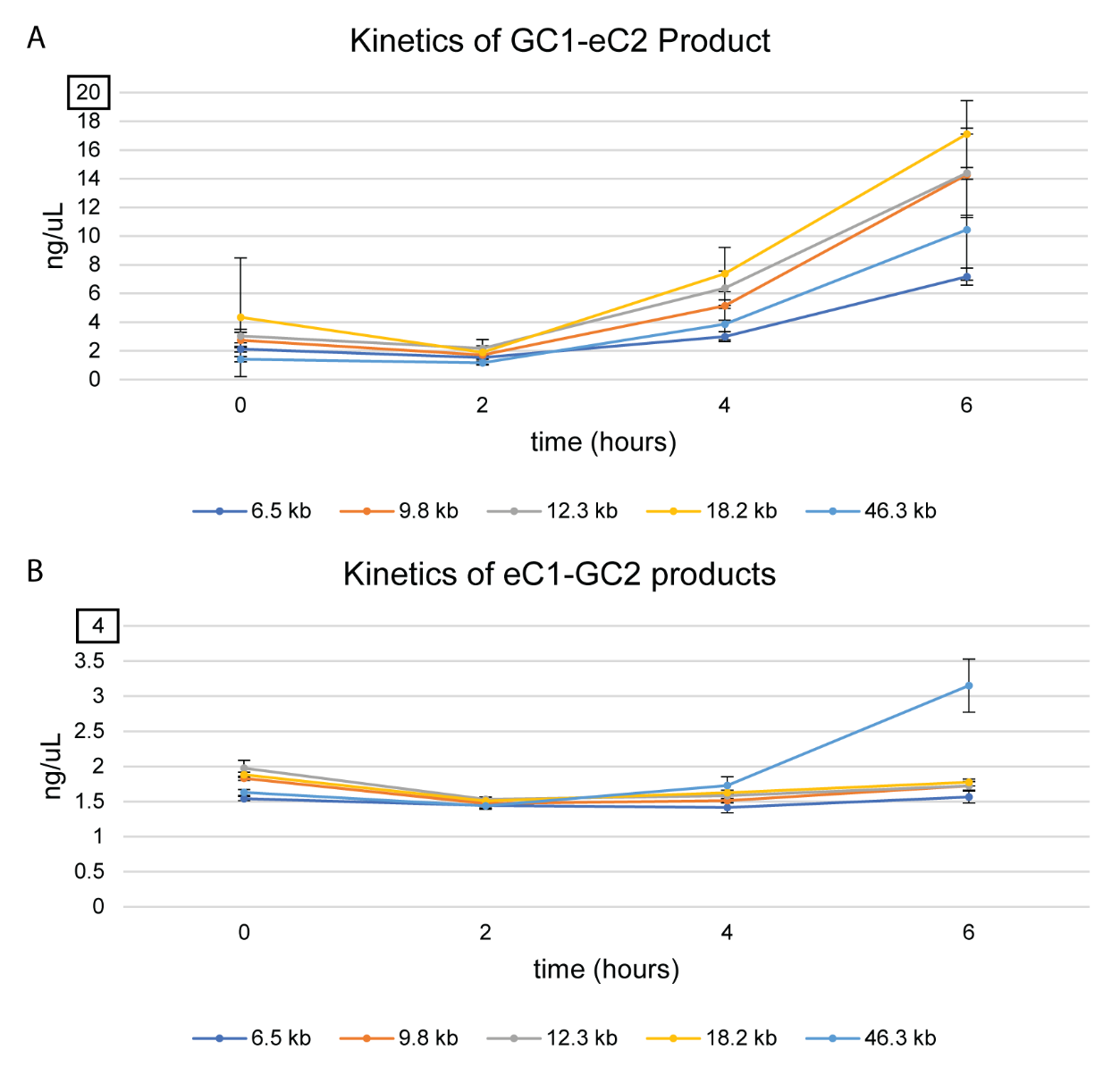


Figure S2. **Recombinant products after dicentric activation, early time points.** Data from Fig. 2. A) Fluorimetry quantitation of recombinant PCR product GC1-eC2 in strains with GALCEN3 inserted 6.5, 9.8, 12.3, 18.2, and 46.3 kb away from the endogenous CEN3, after switching carbon source from galactose to glucose (GALCEN3 activation) and growing for the times indicated. From 0 to 6 hours, the kinetics of GC1-eC2 product generation are similar for all strains. B) Fluorimetry quantitation of PCR product eC1-GC2. Note the difference in y-axis scaling compared to A.


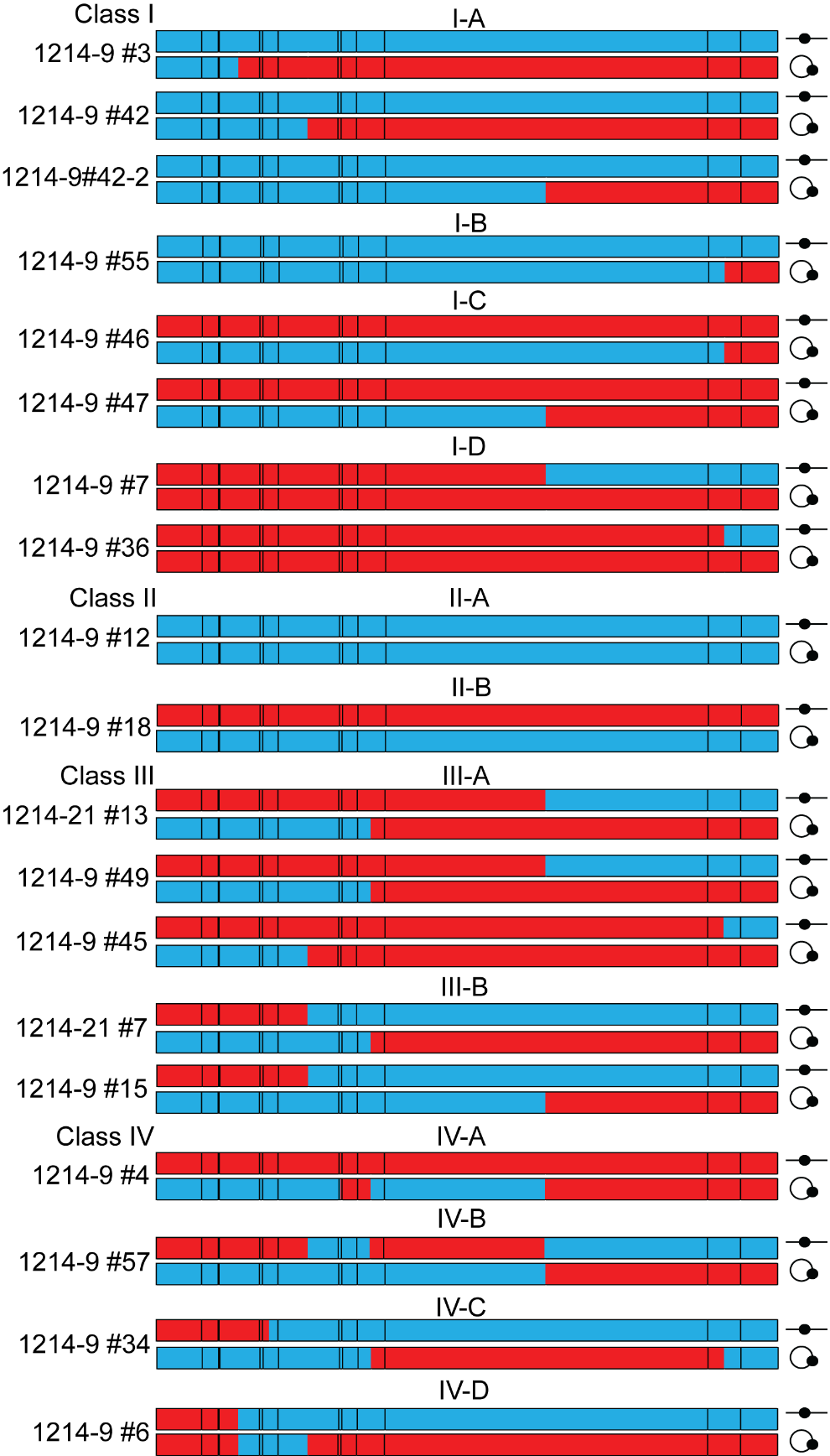


Figure S3. **All sequenced reciprocal recombinants derived from resolution of a dicentric with one s288c centromere and one YJM789 centromere are shown.** The depiction of the recombinants is the same as that used in Fig. 8 with s288c and YJM789 sequences shown in red and blue, respectively. The linear product is at the top of each pair, and the circular product at the bottom. SNPs are shown as short vertical lines within the red and blue rectangles.


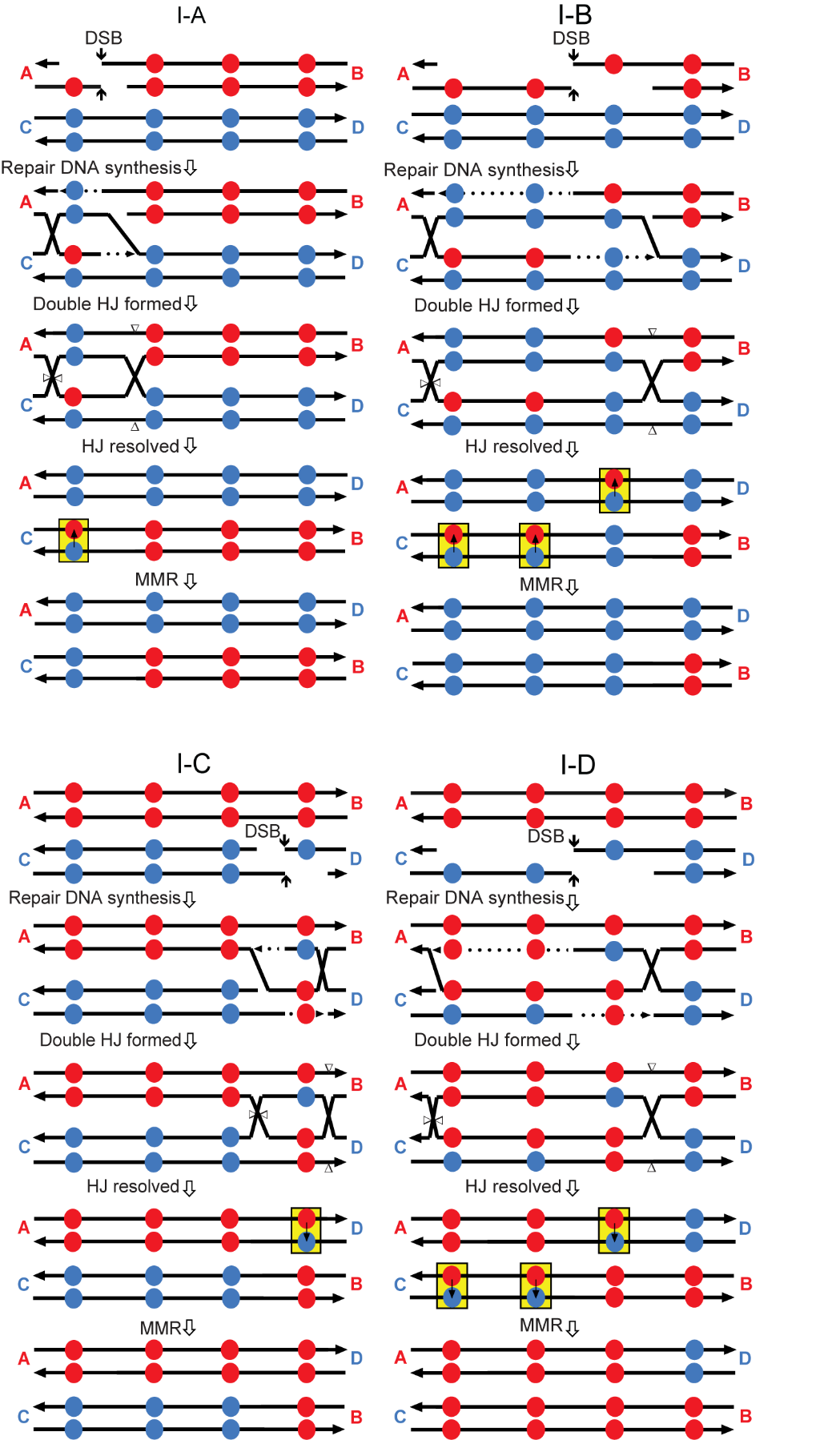


Figure S4. **Recombination intermediates for the generation of recombinant Classes I-A, I-B, I-C, and I-D (Figure S3).** The depiction of the events is described in the legend of Figure 9.


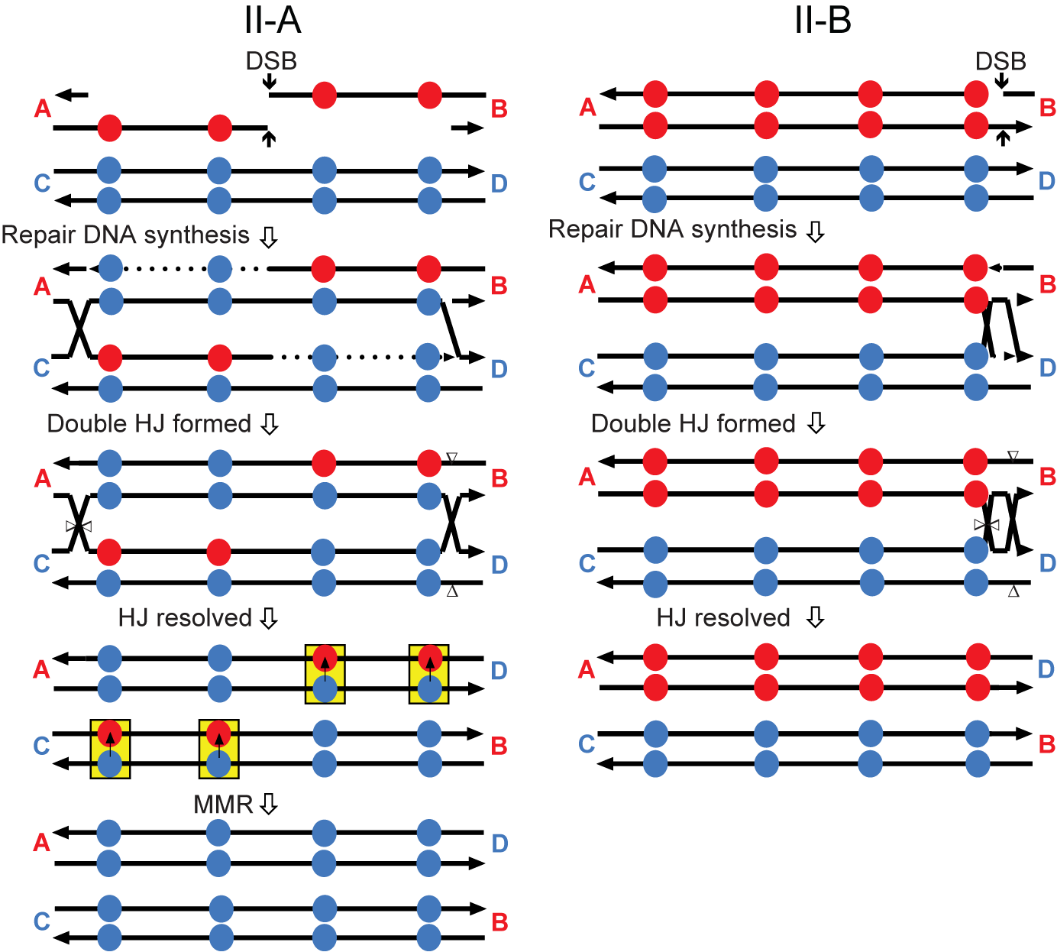


Figure S5. **Recombination intermediates for the generation of recombinant Classes II-A and II-B (Figure S3).** The depiction of the events is described in the legend of Figure 9.


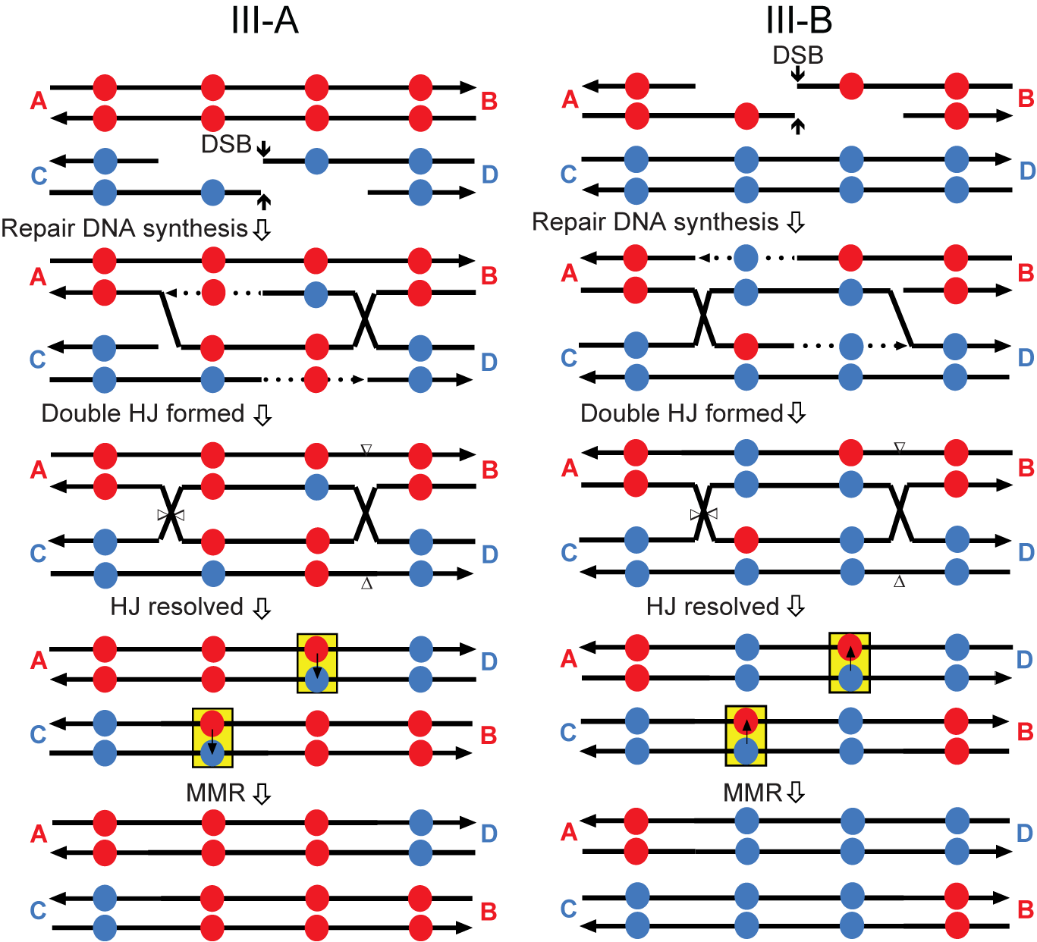


Figure S6. **Recombination intermediates for the generation of recombinant Classes III-A and III-B (Figure S3).** The depiction of the events is described in the legend of Figure 9.


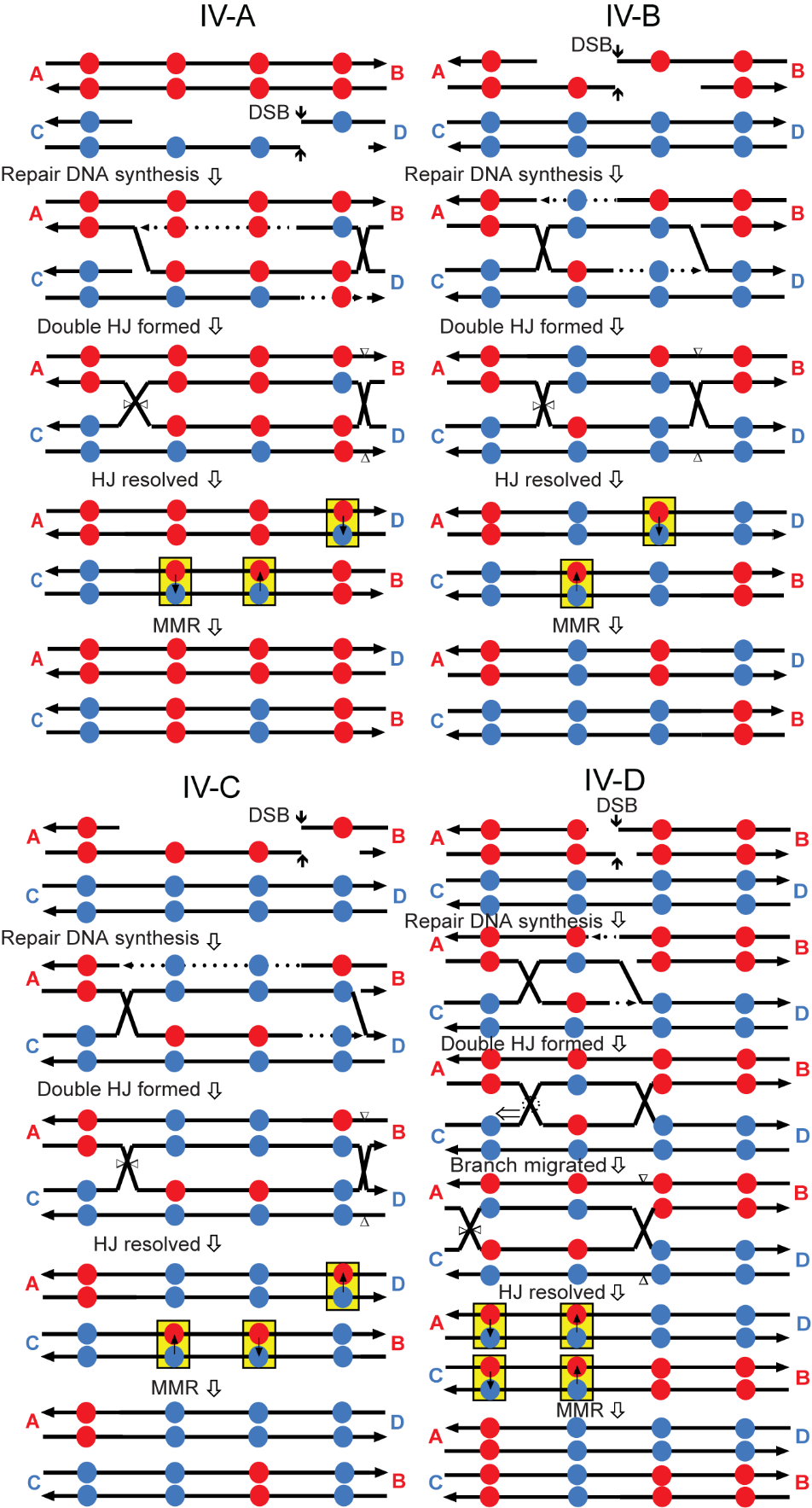


Figure S7. **Recombination intermediates for the generation of the complex recombinant Classes (IV-A)-(IV-D) (Figure S3).** The depiction of the events is described in the legend of Figure 9.

Table S1. **Student’s T-test p values for Figure 2.** Comparing GC1-eC2 to eC1-GC2 for each strain and time point.

|  | 6.5kb | 9.8kb | 12.3kb | 18.2kb | 46.3kb |
| --- | --- | --- | --- | --- | --- |
| 0 | 0.009866863 | 0.000102867 | 5.31737E-05 | 0.093386366 | 0.015520078 |
| 2 | 0.480971805 | 0.038456294 | 1.30464E-07 | 0.219777696 | 0.000286174 |
| 4 | 0.000354896 | 7.94018E-09 | 2.31034E-09 | 6.09957E-08 | 4.2793E-05 |
| 6 | 7.15811E-08 | 4.61138E-10 | 1.64706E-09 | 1.17029E-12 | 2.20651E-05 |
| 24 | 2.33083E-09 | 1.08025E-08 | 7.45911E-13 | 1.53205E-07 | 4.78454E-15 |
| 48 | 3.78257E-14 | 5.99227E-14 | 2.49145E-12 | 4.67869E-11 | 3.55573E-14 |
| 72 | 1.35257E-07 | 1.06647E-07 | 4.43575E-10 | 6.13866E-05 | 2.6056E-07 |

Table S2. **Student’s T-test p values for Figure 2.** Comparing GC1-eC2 to No Product Control.

|  | 6.5kb | 9.8kb | 12.3kb | 18.2kb | 46.3kb |
| --- | --- | --- | --- | --- | --- |
| 0 | 0.045429441 | 0.001219681 | 0.000136766 | 0.248256514 | 0.385033911 |
| 2 | 0.362273205 | 0.054954118 | 3.37342E-05 | 0.308465499 | 0.084618116 |
| 4 | 0.021210138 | 7.36851E-05 | 3.45171E-05 | 0.000239201 | 0.002898415 |
| 6 | 0.000263932 | 1.67401E-05 | 3.53334E-05 | 4.802E-07 | 0.001459114 |
| 24 | 4.62128E-05 | 0.000108239 | 3.64094E-07 | 0.000503198 | 1.01693E-09 |
| 48 | 6.85532E-08 | 7.0246E-08 | 3.99403E-07 | 4.39021E-06 | 2.71724E-09 |
| 72 | 0.000513259 | 3.14023E-05 | 4.04584E-08 | 0.000467598 | 8.09406E-09 |

Table S3. **Student’s T-test p values for Figure 2.** Comparing eC1-GC2 to No Product Control.

|  | 6.5kb | 9.8kb | 12.3kb | 18.2kb | 46.3kb |
| --- | --- | --- | --- | --- | --- |
| 0 | 0.003923167 | 2.24319E-06 | 0.009291333 | 1.25982E-05 | 0.003646529 |
| 2 | 0.139845733 | 0.083699908 | 0.012400877 | 0.005182647 | 0.226432593 |
| 4 | 0.526809522 | 0.011946374 | 0.01543807 | 0.002966253 | 0.099341667 |
| 6 | 0.144181929 | 0.004131097 | 0.009184378 | 0.000685991 | 0.022418297 |
| 24 | 0.028768827 | 0.009819651 | 0.013877615 | 0.000293449 | 1.21038E-06 |
| 48 | 0.030813601 | 0.004769784 | 0.005023855 | 0.000964892 | 4.94549E-06 |
| 72 | 0.009791947 | 0.005928666 | 8.72737E-08 | 0.018525439 | 0.000141344 |

Table S4. **Student’s T-test p values for Figure 3.** Comparing GC1-eC2 to eC1-GC2 for each strain.

| 6.5kb direct | 3.78257E-14 |
| --- | --- |
| 6.5kb inverted | 0.125891075 |
| 9.8kb direct | 5.99227E-14 |
| 9.8kb inverted | 0.009808074 |
| 12.3kb direct | 2.49145E-12 |
| 12.3kb inverted | 0.010121826 |
| 18.2kb direct | 4.67869E-11 |
| 18.2kb inverted | 0.779268294 |
| 46.3kb direct | 3.55573E-14 |
| 46.3kb inverted | 0.036545704 |

Table S5. **Student’s T-test p values for Figure 3.** Comparing GC1-eC2 in direct to GC1-eC2 in inverted strains.

| 6.5kb | 1.08492E-12 |
| --- | --- |
| 9.8kb | 5.28248E-13 |
| 12.3kb | 1.75822E-12 |
| 18.2kb | 1.52557E-09 |
| 46.3kb | 5.2489E-16 |

Table S6. **Student’s T-test p values for Figure 3.** Comparing eC1-GC2 in direct to eC1-GC2 in inverted strains.

| 6.5kb | 0.000639996 |
| --- | --- |
| 9.8kb | 0.002038538 |
| 12.3kb | 0.000102681 |
| 18.2kb | 4.22752E-05 |
| 46.3kb | 9.79675E-11 |

Table S7. **Student’s T-test p values for Figure 3.** Comparing GC1-eC2 to No Product Control.

| 6.5kb direct | 6.85532E-08 |
| --- | --- |
| 6.5kb inverted | 0.050438101 |
| 9.8kb direct | 7.0246E-08 |
| 9.8kb inverted | 0.069390749 |
| 12.3kb direct | 3.99403E-07 |
| 12.3kb inverted | 0.04297098 |
| 18.2kb direct | 4.39021E-06 |
| 18.2kb inverted | 0.057301952 |
| 46.3kb direct | 2.71724E-09 |
| 46.3kb inverted | 0.073324991 |

Table S8. **Student’s T-test p values for Figure 3.** Comparing eC1-GC2 to No Product Control.

| 6.5kb direct | 0.030813601 |
| --- | --- |
| 6.5kb inverted | 0.007569261 |
| 9.8kb direct | 0.004769784 |
| 9.8kb inverted | 0.000739583 |
| 12.3kb direct | 0.005023855 |
| 12.3kb inverted | 0.000226 |
| 18.2kb direct | 0.000964892 |
| 18.2kb inverted | 0.00172619 |
| 46.3kb direct | 4.94549E-06 |
| 46.3kb inverted | 0.054362898 |

Table S9. **Student’s T-test p values for Figure 4A.** Comparing *rad51Δ* GC1-eC2 to WT GC1-eC2.

| 0 | 2 | 4 | 6 | 24 | 48 | 72 |
| --- | --- | --- | --- | --- | --- | --- |
| 2.1407E-05 | 1.6776E-06 | 5.0371E-03 | 9.1814E-01 | 4.7229E-03 | 1.3099E-03 | 1.4488E-10 |

Table S10. **Student’s T-test p values for Figure 4A.** Comparing *rad1Δ* GC1-eC2 to WT GC1-eC2.

| 0 | 2 | 4 | 6 | 24 | 48 | 72 |
| --- | --- | --- | --- | --- | --- | --- |
| 0.06594521 | 0.173823764 | 7.97194E-06 | 2.37079E-06 | 2.13814E-16 | 6.49749E-15 | 1.61453E-15 |

Table S11. **Student’s T-test p values for Figure 4B.** Comparing 9.8 kb *rad51Δ* GC1-eC2 to WT 9.8 kb GC1-eC2.

| 0 | 2 | 4 | 6 | 24 | 48 | 72 |
| --- | --- | --- | --- | --- | --- | --- |
| 0.09314123 | 2.48576E-05 | 0.005246417 | 0.269328655 | 0.065492179 | 0.000984096 | 0.958326547 |

Table S12. **Student’s T-test p values for Figure 4B.** Comparing 9.8 kb *rad51Δ* GC1-eC2 to 46.3 kb *rad51Δ* GC1-eC2.

| 0 | 2 | 4 | 6 | 24 | 48 | 72 |
| --- | --- | --- | --- | --- | --- | --- |
| 0.99214747 | 0.000677584 | 0.312110541 | 0.038914412 | 0.014119766 | 0.818546266 | 0.16785921 |

Table S13. **Student’s T-test p values for Figure 4.** Comparing each value to No Product Control.

|  | 46.3kb *rad1Δ* | 46.3kb *rad51Δ* | 9.8kb *rad51Δ* |
| --- | --- | --- | --- |
| 0 | 0.186957 | 0.005016 | 8.71074E-05 |
| 2 | 0.850845 | 0.007174 | 3.73411E-05 |
| 4 | 0.657998 | 0.00367 | 0.000882868 |
| 6 | 0.135843 | 6.96E-05 | 1.553E-05 |
| 24 | 0.001105 | 1.02E-05 | 0.00061056 |
| 48 | 0.067244 | 4.16E-05 | 6.69312E-05 |
| 72 | 0.01894 | 1.68E-08 | 0.000100319 |

Table S14. **Student’s T-test p value for Figure 4.** Comparing 9.8 kb *rad51Δ* viability to WT.

| 1.82146E-07 |
| --- |

Table S15. **Student’s T-test p value for Figure 4.** Comparing 46.3 kb *rad1Δ* viability to WT.

| 0.034472 |
| --- |

Table S16. **Student’s T-test p values for Figure 5.** HIS4 Popout compared to GC1-eC2 for each time point.

| 0 | 2 | 4 | 6 | 8 | 24 | 48 | 72 | 96 |
| --- | --- | --- | --- | --- | --- | --- | --- | --- |
| 0.06559 | 0.29414 | 0.73669 | 0.01788 | 0.01295 | 0.01062 | 0.01058 | 0.09074 | 0.08132 |

Table S17. **Student’s T-test p values for Figure 5.** HIS4 Popout compared to eC1-GC2 for each time point.

| 0 | 2 | 4 | 6 | 8 | 24 | 48 | 72 | 96 |
| --- | --- | --- | --- | --- | --- | --- | --- | --- |
| 0.03384 | 0.09012 | 0.02813 | 0.02145 | 0.23682 | 0.01608 | 0.00008 | 0.00891 | 0.00521 |

Table S18. **Student’s T-test p values for Figure 5.** GC1-eC2 compared to eC1-GC2 for each time point.

| 0 | 2 | 4 | 6 | 8 | 24 | 48 | 72 | 96 |
| --- | --- | --- | --- | --- | --- | --- | --- | --- |
| 0.24859 | 0.49389 | 0.02537 | 0.00452 | 0.01858 | 0.00723 | 0.00068 | 0.00947 | 0.00644 |

Table S19. **Student’s T-test p value for Figure 6.** 1214-9 compared to WT glu/gal viability.

| 0.0546444 |
| --- |

Table S20. **Student’s T-test p values for Figure 9.** Each strain compared to WT.

|  | WT |
| --- | --- |
| *mph1Δ* | 0.056780817 |
| *mus81Δ* | 1.16845E-08 |
